# Evaluation of 147 Perfluoroalkyl Substances for Immunotoxic and Other (Patho)physiological Activities through Phenotypic Screening of Human Primary Cells

**DOI:** 10.14573/altex.2203041

**Published:** 2022-09-15

**Authors:** Keith A. Houck, Katie Paul Friedman, Madison Feshuk, Grace Patlewicz, Marci Smeltz, M. Scott Clifton, Barbara A. Wetmore, Sharlene Velichko, Antal Berenyi, Ellen L. Berg

**Affiliations:** 1 Center for Computational Toxicology and Exposure, Office of Research and Development, U.S. Environmental Protection Agency, Research Triangle Park, NC, USA; 2 Eurofins Discovery, Burlingame, CA, USA

## Abstract

A structurally diverse set of 147 per- and polyfluoroalkyl substances (PFAS) was screened in a panel of 12 human primary cell systems by measuring 148 biomarkers relevant to (patho)physiological pathways to inform hypotheses about potential mechanistic effects of data-poor PFAS in human model systems. This analysis focused on immunosuppressive activity, which was previously reported as an *in vivo* effect of perfluorooctanoic acid (PFOA) and perfluorooctanesulfonic acid (PFOS), by comparing PFAS responses to four pharmacological immunosuppressants. The PFOS response profile had little correlation with reference immunosuppressants, suggesting *in vivo* activity does not occur by similar mechanisms. The PFOA response profile did share features with the profile of dexamethasone, although some distinct features were lacking. Other PFAS, including 2,2,3,3-tetrafluoropropyl acrylate, demonstrated more similarity to the reference immunosuppressants but with additional activities not found in the reference immunosuppressive drugs. Correlation of PFAS profiles with a database of environmental chemical responses and pharmacological probes identified potential mechanisms of bioactivity for some PFAS, including responses similar to ubiquitin ligase inhibitors, deubiquitylating enzyme (DUB) inhibitors, and thioredoxin reductase inhibitors. Approximately 21% of the 147 PFAS with confirmed sample quality were bioactive at nominal testing concentrations in the 1–60 micromolar range in these human primary cell systems. These data provide new hypotheses for mechanisms of action for a subset of PFAS and may further aid in development of a PFAS categorization strategy useful in safety assessment.

## 1 Introduction

Per- and polyfluoroalkyl substances (PFAS) are a large class of chemicals in widespread use for diverse applications in commerce resulting in significant presence in the environment. Extensive studies of several of the highly produced members of the class have demonstrated potential for adverse health consequences to humans as well as highly pervasive and persistent exposures (Wang et al., 2017; Cousins et al., 2019). These findings have led to major restrictions on the manufacture and use of several PFAS internationally, in particular perfluorooctanoic acid (PFOA) and perfluorooctanesulfonic acid (PFOS), with consequential development of new PFAS as commercial alternatives (ECHA, 2014; UN, 2020; OECD, 2015; EPA, 2000, 2020). Due to the high number and structural diversity of PFAS, the OECD now defines PFAS as substances with a minimum of one perfluorinated carbon, which results in a list of PFAS that may approach 40,000 substances, with more stringent definitions of PFAS structures resulting in shorter lists in the thousands (Williams et al., 2022; OECD, 2021). The Organization of Economic Cooperation and Development (OECD) has compiled a list of 4,730 unique CAS Registry numbers for PFAS that may have been on the global market (OECD, 2018). Minimal to no toxicity information is available on PFAS and degradation products that span diverse chemical structural features beyond PFOA and PFOS. Given the large numbers of substances requiring assessment, use of new approach methodologies (NAMs) allowing more rapid testing and evaluations of substances may be useful for filling data gaps.

A suite of NAMs covering a range of known and suspected PFAS adverse effects is being used to test a diversity of PFAS structural categories as part of the EPA’s PFAS Action Plan (Patlewicz et al., 2019; EPA, 2019). Results from this large suite of NAMs may provide information relevant to the first tier of information for evaluating the PFAS hazard within structural categories, following the plan described in the National PFAS Testing Strategy (EPA, 2021). One component of initial NAM screening is evaluation of effects of PFAS on immune function, a potential adverse effect *in vivo* noted for both PFOA and PFOS characterized by suppression of T cell-dependent antibody production and reduced antibody response to vaccinations (NTP, 2016; EFSA, 2020). Mechanisms underlying these effects are not known.

Here we report testing results of 147 PFAS substances in a phenotypic screening platform of primary human cell co-culture systems, the BioMAP^®^ Diversity Plus panel, used to model complex tissue and disease biology of organs (vasculature, immune system, skin, lung) and general tissue biology. Use of the BioMAP panel of human primary cell systems as (patho)physiologically relevant screening assays for evaluating adverse effects was previously demonstrated through testing pharmaceuticals and clinical candidates as well as environmental chemicals in the EPA’s ToxCast program (Kleinstreuer et al., 2014; Berg et al., 2006; Singer et al., 2019). The BioMAP Diversity Plus panel includes 12 assays encompassing 148 endpoints particularly enriched with capabilities to detect modulators and effectors of vascular and immune biology. This panel has been extensively used in pharmaceutical and consumer products research for characterization of product candidates (O’Mahony et al., 2018; Betts et al., 2018; Hammitzsch et al., 2015; Shah et al., 2017; Simms et al., 2021; Singer et al., 2019). Of note for testing PFAS is the inclusion of the BioMAP T cell activation system (SAg) measuring multiple endpoints modulated by a cocktail of superantigens, and the B and T cell autoimmunity assay (BT) for T cell-dependent B cell activation and antibody production as key modulators of the innate and adaptive immune response, respectively. Additional assays include models of vascular inflammation, monocyte activation, lung inflammation and fibrosis, cardiovascular inflammation, and wound healing.

A representative set of 147 PFAS was selected for testing using criteria that included: characterizing the OECD PFAS chemical database into structural categories and prioritizing them based on considerations such as whether categories contained PFAS that were of interest to the U.S. Environmental Protection Agency (EPA); whether they contained PFAS with existing *in vivo* data that could be useful in developing a read-across approach for data gap-filling; whether the PFAS were technically feasible to test based on physicochemical considerations (solubility/volatility); and whether the PFAS helped to represent structural diversity among this group of chemicals (Patlewicz et al., 2019). As our primary objective was to examine potential immunosuppressive activity of the PFAS, we included four known immunosuppressants (azathioprine, methotrexate, dexamethasone, and cyclosporine A) covering different mechanisms of immunosuppressive action in the test set with 147 PFAS. Distinct signatures in the BioMAP panel for several of these agents including methotrexate, dexamethasone, and cyclosporine A have been previously reported (O’Mahony, et al., 2018; Berg et al., 2013). We tested the hypothesis that PFAS with mechanisms of action like these reference immunosuppressive compounds would have similar response profiles in the cell systems relevant to immunosuppression. Our general experimental approach included testing PFAS at four concentrations, ranging from approximately 0.06 to 60 micromolar, in order to minimize influence from confounding effects of polypharmacology resulting from qualitatively different activities at higher concentrations. Additionally, we compared response profiles for all PFAS with an existing database of responses for the BioMAP assays to identify other potential mechanisms of activity for this diverse chemical family in an effort to generate hypotheses about the mechanism(s) of action for data-poor PFAS in this 147 PFAS chemical library.

## 2 Materials and methods

### 2.1 Chemical library

PFAS were selected from a comprehensive database of 4,730 PFAS based on structural category, interest to the EPA, ability to be commercially procured, solubility in dimethyl sulfoxide (DMSO), and structural diversity to support development of read-across (Patlewicz et al., 2019). PFOA and PFOS were included in the 147 PFAS as test chemicals (not as separate controls). The PFAS used in this study are listed in Table S1^1^, along with their average mass (g/mol), the analytical quality control grades on the solubilized chemical samples (stock samples), the final concentrations used in screening in the BioMAP assays, and the structural categories based on ChemoType ToxPrints (Yang, C. et al., 2015) that have been developed specifically to describe the structural features present in the PFAS library. The full PFAS chemical testing library and associated PFAS selected for testing are also available as lists on the EPA CompTox Chemicals Dashboard^2^. All were procured by Evotec (US), Inc. (Branford, CT) under contract to the EPA (Contract #EP-D-12-034). Substances were solubilized in 100% DMSO at library stock concentrations of 30 mM if achievable without visible precipitation. One chemical, ammonium perfluoro-2-methyl-3-oxahexanoate (GenX), was solubilized in H_2_O as it was known to be unstable in DMSO (Gaballah et al., 2020; Liberatore et al., 2020). Sequential dilutions in DMSO to 10 mM were used, if necessary, to achieve soluble stock solutions without precipitation. Four additional samples of reference chemicals with known immunosuppressant activity, cyclosporin A (Light Biologicals), azathioprine (Sigma Chemical Company), dexamethasone sodium phosphate (Sigma Chemical Company), and methotrexate (ThermoFisher Scientific), were also procured by Evotec, solubilized in DMSO, and included in the test set. Stock solutions were stored sealed and frozen at −80°C and duplicate samples shipped in blinded format to Eurofins Discovery Services (St. Louis, MO) for screening in the BioMAP Diversity Plus panel under EPA contract 68HE0D18D0002.

### 2.2 Library quality control analysis

DMSO stock solutions were used as this is a universal solvent having good general small-molecule solvating capability and compatibility with high-throughput biological assays (Popa-Burke et al., 2004). Evolving knowledge of instability of certain PFAS in DMSO (Liberatore et al., 2020; Zhang, C. et al., 2021) led to a stock quality and stability evaluation across the PFAS stocks employed in this work. Full mass spectrometry (MS) scans were conducted to determine if each PFAS parent structure was present in the stock solution. A binary pass/fail grade was assigned to each stock, where stocks failed if no chemical was detected and/or if significant degradation was evident (Smeltz et al., in preparation). Informational flags were also assigned to describe additional characteristics of the stocks (see Tab. S1^1^ for all analytical grades and flags).

For those PFAS samples undergoing evaluation using liquid chromatography (LC) separation, a Waters Corporation (Milford, MA) ACQUITY I Class ultra-high-performance LC coupled to an Xevo TQ-S micro-MS was used in RADAR mode, rapidly switching between multiple reaction monitoring mode (MRM) and MS full scan acquisition, to monitor each PFAS while also evaluating any interferences. To grade each compound, MS full scan data were reviewed for the presence of parent mass, while a confirmatory check of the PFAS response was performed with the acquired MRM data.

For chemicals analyzed by gas chromatography (GC)-MS, full scans were generated on an Agilent (Palo Alto, CA) 6890/5973N GCMS across three ionization modes (electron impact, and negative and positive chemical ionization). Chromatograms were evaluated for peak presence and co-occurrence across ionization modes. Spectra were extracted, background subtracted, and evaluated to confirm chemical identity using NIST 17 (National Institute of Standards and Technology) database spectra for comparison when available. It was beyond the scope and resources available to employ additional technologies (e.g., nuclear magnetic resonance spectroscopy) or detection strategies to further characterize this large collection of diverse perfluorinated chemicals, and as such, pass/fail grades were based on the LC and GC methods described herein and are intended to provide context to the bioactivity screening results reported.

### 2.3 Conducting the BioMAP assays

The following subsections describe how the BioMAP assays were conducted following the experimental sequence of events: how the human primary cell co-cultures were sourced and cultured; the BioMAP systems created from these co-cultures; how the BioMAP systems were stimulated to make (patho)physiologically relevant models; how chemical exposures were conducted for chemical screening; and how biomarker endpoints were measured (including indicators of cell proliferation/viability). All studies were performed under contract with Eurofins Discovery using the BioMAP^®^ Diversity Plus^®^ panel (previously known as BioSeek assays, or BSK, within prior work with the ToxCast program).

### 2.4 Human primary cell co-culture

Use of human primary cell types followed the guidelines for human subjects research under United States Department of Health and Human Services (HHS) human subjects regulations (45 CFR Part 46). Preparation and co-culture of human primary cell types and methods for the systems were as previously described (Melton et al., 2013; Shah et al., 2017; Kunkel et al., 2004a). Human umbilical vein endothelial cells (HUVEC) and human neonatal foreskin fibroblasts (HDFn) were cultured according to the supplier’s (Lonza, Inc., Allendale, NJ) recommendation and plated to confluence for all endpoints other than proliferation endpoints (see below). Primary human bronchial epithelial cells (Cell Applications, Inc., San Diego, CA), arterial smooth muscle cells, adult lung fibroblasts (Lonza, Inc., Allendale, NJ), and keratinocytes (Cambrex, Inc., East Rutherford, NJ) were cultured according to methods recommended by the commercial suppliers and plated to confluence for all endpoints other than proliferation endpoints (see below). Some of the assay systems employed do contain low amounts of fetal bovine serum (FBS) to ensure cell health. The BF4T, BE3C, and KF3CT systems contain no FBS; the HDF3CGF system contains 0.13% FBS; the 3C, 4H, LPS, SAg and Mphg systems contain 2% FBS; the CASMC3C system contains 5% FBS; and the BT system contains 10% FBS.

Peripheral blood mononuclear cells (PBMC) were prepared from buffy coats from normal human donor blood samples (Kunkel et al., 2004b) obtained via BioIVT (HUMAN-LMX100-0001129) to create a pool of human donors to minimize variability in assay responses for the specified biomarkers measured. PBMC from different donors were banked, then cells from 3–5 donors were pooled and added to wells at the time of assay initiation. Though different pooled donor sets may be used, stimulation is required to observe activation in these assays (unpublished observation). Donor pools may differ in terms of donor demographics, and this may contribute to variability in the responses; as such, normalization of the data to neutral control wells, positive control performance, and assay qualification requirements ensure that data can be compared between experiments. CD20^+^ B cells and CD14^+^ monocytes were obtained from All Cells, Inc., Emeryville, CA. Macrophages were prepared by culturing CD14^+^ monocytes in M-CSF (50 ng/mL) for 7 days.

All primary human cells utilized in this work were obtained via commercially available sources and were used at early passage (≤ P4) or without passaging (in the case of PBMC and B cells) to minimize adaptation to cell culture and preserve physiological signaling responses.

### 2.5 BioMAP systems

Primary human cell types used in BioMAP systems and their stimuli included the following: 3C System (HUVEC/IL-1β, TNFα and IFNγ), 4H System (HUVEC/IL-4 and histamine), LPS System (PBMC and HUVEC/LPS), SAg System (PBMC and HUVEC/TCR ligands), BT System (CD19^+^B cells and PBMC/anti-IgM + TCR ligands), BE3C System (bronchial epithelial cells/IL-1β, TNFα and IFNγ), BF4T System (bronchial epithelial cells and human dermal fibroblasts/TNFα and IL-4), HDF3CGF System (human dermal fibroblasts/IL-1β, TNFα, IFNγ, EGF, basic-FGF and PDGF-BB), KF3CT System (keratinocytes and dermal fibroblasts/IL-1β, TNFα and IFNγ), CASM3C System (coronary artery smooth muscle cells/IL-1β, TNFα and IFNγ), MyoF System (differentiated lung myofibroblasts/TNFα and TGFβ), Mphg System (HUVEC and macrophages/TLR2 ligands) (Tab. S2^1^).

### 2.6 BioMAP systems stimuli

Assays were initiated by addition of chemical samples for 1 h followed by addition of appropriate stimuli. Assay plates were then incubated for 24 h unless otherwise indicated. The MyoF system was stimulated for 48 h, and the BT system was stimulated for either 72 h (soluble readouts) or 6 d (for measurement of secreted IgG). Concentrations of stimuli were as follows: cytokines (IL-1β, 1 ng/mL, Peprotech 200–01B; TNFα, 5 ng/mL, Peprotech 300–01A; IFNγ, 20 ng/mL, Peprotech 300–02; IL-4, 5 ng/mL, 200–04), activators (histamine, 10 μM, Sigma H7125; SAg, 20 ng/mL or LPS, 2 ng/mL, Sigma L7770), growth factors (TGF-β, 5 ng/mL, R&D Systems 240-B/CF; EGF, Peprotech AF-100-15; basic-FGF, ThermoScientific 13256029; PDGF-BB, 10 ng/mL, Peprotech 100–14B; Zymosan, 10 μg/mL, Invivogen tlrl-zyn; Anti-IgM, 500 ng/mL). Superantigens (SAg), staphylococcal enterotoxin B (SEB) and toxic shock syndrome toxin-1 (TSST-1) (staphylococcal enterotoxin F) from *Staphylococcus aureus*, and lipopolysaccharide (LPS) from *Salmonella enteritidis* were obtained from Sigma. The number of lymphocytes or macrophages added to the SAg, LPS, BT and Mphg systems were as follows for 96-well format: B cells (2.5 × 10^4^), PBMC (7.5 × 10^4^ cells/well for LPS and SAg systems or 2.5 × 10^4^ cells/ well for BT system) or macrophages (7.5 × 10^4^ cells/well). After stimulation, plates and supernatants were harvested and biomarkers quantitated by ELISA and other methods (see Biomarker endpoint measurements).

### 2.7 Chemical screening

Chemical samples (defined as the PFAS, reference chemicals, and controls, each solubilized in the appropriate solvent) were screened at indicated concentrations in a single well per biomarker endpoint. Each sample was screened in duplicate in an independent and blinded format, i.e., the identity of the chemical in the sample was unknown to the experimenter and only revealed when data were analyzed. Chemical samples were added 1 h before stimulation of the cells and were present during the subsequent 24 h - 6 d stimulation period. Final DMSO concentration in each assay well was < 0.1%. Colchicine (a cytotoxic chemical at 3.3 μM) and non-stimulated control samples were included on every plate, for all assays in the BioMAP panel. Eight replicates of vehicle control (DMSO at 0.1%) were included on each plate.

### 2.8 Biomarker endpoint measurements

The levels of cell surface (or secreted, indicated by the prefix “s”) biomarker endpoints were measured by ELISA as described (Shah et al., 2017; Melton et al., 2013). Overt cytotoxicity to cells in confluent adherent cultures (all systems other than the BT system) was assessed by measuring total protein levels using sulforhodamine B (SRB) staining (Gerçel-Taylor et al., 2001) in parallel cultures at the time of biomarker measurements. These are indicated as SRB endpoints. For proliferation assays for adherent cell types, individual cell types are cultured at sub-confluence and relative cell numbers quantified by SRB staining at time points optimized for each system (48 h: 3C and CASM3C systems; 72 h: BT and HDF3CGF systems; 96 h: SAg system). SRB was performed by staining cells with 0.1% SRB after fixation with 10% TCA and reading wells at 560 nm.

Viability and proliferation of PBMC (T cells) was quantified by Alamar Blue reduction (Ahmed et al., 1994) for the SAg and BT systems. For PBMC viability (referred to as PBMC Cytotoxicity within the assay endpoint names), cells were plated (75,000/well in a 96-well plate) and then chemical samples added for 1 h before addition of activators, SEB and TSST-1 (20 ng/mL final concentration each). Cells were incubated for 90 h. Then, Alamar Blue (20 μL/well) (Invitrogen, Cat #DAL1100) was added for 6 h, and the plates were read with a fluorescence microplate reader at 546/580 nm (excitation/emission). For PBMC proliferation, cells were plated and activated as above but incubated for only 16 h prior to addition of Alamar Blue. After 6 h, plates were read as described above.

### 2.9 Data processing

Measurement values for each well (one biomarker per well) were divided by the mean value from 8 DMSO control samples (from the same plate) to generate a ratio. GenX was the only PFAS solubilized in water. However, the final concentration of DMSO was consistent for all substances tested, as DMSO was added to the GenX-treated wells to match the other chemical-treated wells in the BioMAP system for which the chemical samples were solvated in DMSO. The GenX-treated wells were then normalized to the same DMSO-treated control wells as all other chemicals in the set. All ratios were then log_10_ transformed. Historical controls are the log_10_-ratios of DMSO control wells that are collected over time (23 experimental runs collected over 2 years). Significance prediction envelopes were calculated for historical controls, and the 95% envelope was employed. Overtly cytotoxic compounds were identified as generating profiles with one or more of the following readouts below the indicated thresholds: SRB < −0.3, PI or PBMC Cytotoxicity < −0.3 in one or more systems.

### 2.10 Lowest effective concentration determination

This project proceeded as part of the EPA ToxCast program, and as such, data processing with the ToxCast Data Pipeline (tcpl, v2.1.0) was employed to manage these data. The data were stored in the ToxCast database, invitrodb. Tcpl was also used to codify how the lowest effective concentrations of PFAS in the BioMAP panel were identified. Lowest effective concentration for these data was defined as the concentration where activity was greater than the threshold cutoff for a positive. This threshold cutoff was defined as the maximum of either three times the median absolute deviation of wells that represented baseline or a log_10_(1.2)-fold change, as described in detail in the next subsection. ToxCast data are made publicly available via releases of the ToxCast database and in the CompTox Chemicals Dashboard^5^.

#### Detailed tcpl procedure

The transformed ratios for the 12-assay BioMAP panel were received by the EPA and loaded into the ToxCast database, invitrodb (released in invitrodb version 3.5 in 2022) under the BioSeek assay source identifier, abbreviated as BSK. BSK was used for continuity in invitrodb and in public versions of ToxCast data despite more recent changes in the name and ownership of the assay technology (now owned by Eurofins Discovery and referred to as BioMAP systems). Loading these data to the ToxCast database is multi-purpose; primarily, it makes the data publicly accessible as log_10_-fold change, enabling these data to inform other, future analyses. Processing with the ToxCast Pipeline (tcpl) also provides estimates of potency for bioactivity across all assay endpoints that comprise the BioMAP suite (see Fig. 2).

Data were processed using R library tcpl^6^ (v2.1.0) (Filer et al., 2017) using methods to identify lowest observable effect concentration for BSK data rather than curve-fitting these data as is done for most other ToxCast data. Invitrodb is comprised of data stored at various levels, which are described in detail here for the BioMAP panel. Level 0 stored the “raw” response values (the transformed ratios) for the 148 assay components, and Level 1 processing set concentration and replicate indices from this input. At Level 2, no additional data transformations were necessary since the data were pre-processed by the vendor. Level 3, typically a normalization step in tcpl, applied no additional normalization except to invert data for the loss of signal (_down) endpoints. This inversion was done so that all assay endpoint response profiles could be represented in the positive direction, with values increasing from a baseline of zero, as is customary for the ToxCast program in the current version of invitrodb. At Level 4, the baseline median absolute deviation (bmad) was calculated using the responses at the two lowest test compound concentrations across each endpoint, which were intended to represent a conservative estimate of baseline sampling variability. Though curve-fitting models were applied via default functionality of tcpl at Level 4, the curve-fitting procedure for BSK data is less quantitatively informative than the lowest effect concentration or concentration where activity was greater than the threshold cutoff (coff) for a positive. As such, the curve-fitting information for BSK should be disregarded. A positive hit call (hitc = 1) was assigned if the replicate responses at any concentration exceeded the coff, which was the maximum of three times baseline median absolute deviation (3*bmad) or log_10_(1.2), as determined on an assay endpoint basis. The Level 5 method, loec.coff, was applied to identify the lowest observed effect concentration for samples with positive hit calls that meet the criteria described. The Level 6 caution flag information for BSK should be disregarded as curve-fitting models were not used for potency estimation. The relevant output data from tcpl (Level 5 information) are provided in Table S3^7^.

### 2.11 Unsupervised clustering

Chemical responses (log_10_ fold-change) across all assay endpoints at individual concentrations and replicate were clustered using the Self-Organizing Map algorithm from Partek Genomics Suite (v7.17.1222) (St. Louis, MO). This unsupervised clustering using the 147 PFAS and the immunosuppressive reference chemicals was intended to group the chemicals into clusters with the greatest similarity in their responses across the BioMAP assay suite and further to understand if any of the PFAS response profiles are similar to the response profiles of the immunosuppressive reference chemicals.

### 2.12 Similarity search analysis

Profile similarities were evaluated for each compound/concentration pair across the BioMAP suite to chemicals, drugs, and cosmetics previously screened in the BioMAP assay suite. Profiles are simply defined as the set of responses across all screened endpoints in BioMAP for a given chemical sample. This analysis was intended to support hypotheses for potentially shared biological targets between data-poor PFAS and previously screened chemicals with known biological targets. This similarity search analysis relies on inference to data generated using BioMAP for a diverse set of chemicals (Berg, 2019; Berg et al., 2006, 2013). For this analysis of profile similarities, overtly cytotoxic compound profiles were removed, as this gives results that confound the interpretation of mechanistic similarity. As described in the toxicity signature analysis section below, some overtly cytotoxic chemicals affect multiple cell types, and particularly cause cytotoxicity of HUVEC stimulated under inflammatory conditions, which is a profile preferentially associated with chemicals that cause acute toxicity *in vivo*. Similar profiles in BioMAP were identified by positive Pearson correlation. Profile pairs having the same target mechanisms typically have Pearson correlations with r > 0.7 (Berg et al., 2013). Here we report the top 10 most similar profiles with Pearson correlations of r > 0.6 to capture a wider range of potential mechanistic hypotheses.

### 2.13 Toxicity signature analysis

Toxicity signatures are made up of 2–5 biomarker activities in the BioMAP suite that have been associated with an increased risk of certain toxicity effects *in vivo*. Biomarker activity patterns for nine BioMAP toxicity signatures (acute toxicity, immunosuppression, skin irritation, liver tox, organ tox, skin rash, skin sensitization, thrombosis, and vascular toxicity) were developed by data mining the BioMAP Reference Database to identify common activities between the profiles of drugs with the same reported clinical adverse effects or *in vivo* effects (e.g., acute toxicity) (Berg, 2019). Knowledge of key activities identified in BioMAP profiles was combined with clinical data to determine which of the biomarker activities is associated with a positive and negative impact on the particular biology involved. The strength of clinical associations was tested by comparing this biomarker pattern against the BioMAP Reference Database to determine consistency in the presence or absence of the signature across other drugs with reported adverse effects. While these alerts may not represent all possible mechanisms by which these outcomes occur (showing greater accuracy than sensitivity), the compounds used to define toxicity signatures allow mechanistic insight into underlying events regulating these clinically reported side effects. Details of each toxicity signature, including the drugs or chemicals used to identify the signature, the key biomarker readouts, and mechanisms associated with each signature, are described in detail elsewhere (Berg, 2019).

Evaluation of the presence or absence of toxicity signatures within the BioMAP profile of the tested agent was performed at each concentration. Concentrations are listed if the toxicity signature for the indicated alert was detected at two or more concentrations (indicated as ≥ the lowest concentration), or at the top concentration (concentration is listed without a symbol). Not detected (nd) indicates that the alert signature was not detected at any of the concentrations tested. Not assessed (NA) indicates that the alert signature could not be assessed at the concentrations tested (for example, if the chemical sample was overtly cytotoxic at all concentrations tested).

The process for evaluating profiles for the presence of toxicity signatures is stepwise. Each compound/concentration pair is first assessed for overt cytotoxicity in adherent cell types in all systems that measure total protein (SRB readouts). Profiles are flagged for acute toxicity if three or more SRB endpoints have Log10Ratio values ≤ −0.3 and one or more of the endpoints is in an endothelial cell-containing system (3C, 4H, LPS or Mphg system). Mechanisms associated with the acute toxicity signature include inhibition of protein synthesis, RNA synthesis, and Na^+^/K^+^ ion transport. Sample concentrations for which acute toxicity was flagged were not evaluated further. Profiles remaining were next evaluated for the liver toxicity signature. If the 3C:SRB endpoint had Log10Ratio value ≤ −0.3, the profile was flagged for liver toxicity and then analyzed for the remaining toxicity signatures (Berg, 2019). Mechanisms associated with the liver toxicity signature include inhibitors of vacuolar-type ATPase (V-ATPase), phosphoinositide kinase, FYVE-type zinc finger containing (PIKfyve) and smoothened (Smo). Profiles that showed decreased proliferation of endothelial cells, outside the 95% historical control envelope and with an effect size of 20% (Log10Ratio < −0.1) but that were not cytotoxic in this system (3C:SRB > −0.3), were flagged for the organ toxicity signature. Mechanisms associated with this signature include inhibitors of DNA replication and microtubule function. Profiles that had decreased proliferation of T cells (SAg:Proliferation), outside the 95% historical control envelope and with an effect size of 20% (Log10Ratio < −0.1), or decreased levels of IgG and B cell proliferation (BT:sIgG, BT:Proliferation) or were cytotoxic to PBMC (SAg:PBMC Cytotoxicity or BT:PBMC Cytotoxicity having Log10Ratio < −0.3) were flagged for the immunosuppression signature. Mechanisms associated with this signature include inhibition of mammalian target of rapamycin (mTOR), calcineurin, JAK3, hsp90, NFAT and DNA proliferation (Berg, 2019). All other signatures were tested at non-cytotoxic concentrations. Profiles that had increased levels of tissue factor (TF) in the BioMAP 3C, outside the 95% historical control envelope and with an effect size of 20% (Log10Ratio > 0.1) but that were not cytotoxic in this system (3C:SRB > −0.3), were flagged for the thrombosis signature. Target mechanisms associated with the thrombosis signature include mTOR, AhR, V-ATPase, lysosomal function, CYP17A, PKC NOD2, estrogen receptor, H1R, HIF-1alpha, thyroid hormone receptor, OSM R (Berg et al., 2015). Profiles that showed increased levels of PGE2 in the BioMAP LPS system, outside the 95% historical control envelope and with an effect size of 20% (Log10Ratio > 0.1), and increased or unchanged levels of TNFα (LPS:sTNFα) were flagged for the skin irritation signature. Target mechanisms associated with this signature include RAR/RXR, AhR, and VDR. Profiles that had decreased levels of Collagen III in the BioMAP HDF3CGF system, outside the 95% historical control envelope and with an effect size of 20% (Log10Ratio < −0.1), were flagged for the skin sensitization signature. Target mechanisms associated with this signature include RAR/RXR, PKC, JNK, and prostaglandin receptors. Profiles that showed increased levels of VCAM-1 in the BioMAP HDF3CGF system, outside the 95% historical control envelope and with an effect size of at least 20% (Log10Ratio > 0.1), were flagged for the skin rash (MEK-associated) signature. Target mechanisms associated with this signature include MEK, p38MAPK, IL-1R, IL-4R, Tweak receptor and IFNα/β. Profiles that had increased levels of acute phase serum amyloid A (SAA) in the BioMAP CASM3C system, outside the 95% historical control envelope and with an effect size of 20% (Log10Ratio > 0.1), were flagged for the vascular toxicity signature. Target mechanisms associated with this signature include MEK, GR, MR, HDAC and IL-6R.

The presence of a toxicity signature for a given chemical (in this case, a PFAS) does not necessarily imply that it will cause the corresponding toxicity *in vivo* but rather that further studies may be of interest to confirm a potential association or associated mechanism-of-action.

### 2.14 Assay acceptance criteria

The BioMAP platform generates multi-parameter data sets for each compound tested. Assays are plate-based, and performance is assessed by positive and negative controls for each assay. Negative controls included buffer and solvent (e.g., DMSO). For stimulated systems, positive controls included the non-stimulated condition (non-stim) and a positive control sample (colchicine). Data acceptance criteria were based on plate performance (%CV of negative control wells) and the performance of positive controls across assays with a comparison to historical controls. The performance of each BioMAP system in a given assay was evaluated using the Pearson statistic for the positive control, calculated individually for each assay compared to the positive control reference dataset. This test, the QA/QC Pearson test, was performed by first establishing the 1% false negative Pearson cutoff from the positive reference dataset. The process was iterated through each profile in the positive control reference dataset, calculating Pearson values between this profile and the mean of the rest of the profiles in the dataset, so the number of Pearson values calculated was the number of profiles in the reference dataset. The value at the one percentile of all Pearson values calculated was set as the 1% false negative Pearson cutoff. If the Pearson between a new positive control profile and the mean of positive control reference profiles exceeded this 1% false negative Pearson cutoff, then these plates passed the test. Assays were accepted when the positive control passed the Pearson test and 95% of plates had % CV < 20%. Plots show all points for individual samples unless indicated otherwise.

### 2.15 Quality assurance

Eurofins Discovery ensured the quality of all internal testing, operations, and data released using a comprehensive quality management system (QMS). The QMS was implemented through detailed standard operating procedures (SOPs) within the documentation management system that is controlled and maintained by the Quality Assurance Unit (QAU) at Eurofins Discovery.

## 3 Results

### 3.1 Analytical QC

PFAS stock solutions were prepared in DMSO for bioactivity testing and analyzed by appropriate analytical chemical procedures to determine if the expected structure was present. Results are summarized in Table S1^1^ along with flag definition/assignment and functional category assignment. There were some stock failures with no parent detected for PFAS with predicted low boiling points and high vapor pressure. These were assigned the flag “Fns.” A few others failed due to degradation in DMSO, including ammonium perfluoro-2-methyl-3-oxahexanoate (DTXSID70880215; GenX) and were assigned the flag “Fde.” The water stock of GenX passed the stock quality evaluation. Analytical results were used to help with interpretation of bioactivity testing data, not as a decision criterion regarding whether to test or report data. For PFAS samples with analytical quality control failures but lacking bioactivity, the lack of bioactivity may be due to a lack of presence of the target PFAS in the assay; for PFAS samples with analytical quality control failures with bioactivity, the bioactivity may have resulted from uncharacterized PFAS degradants or metabolites at unknown concentrations.

### 3.2 Understanding the bioactivity of samples

Bioactivity testing was conducted on 147 PFAS plus four reference immunosuppressant compounds at four concentrations in 12 co-culture cell systems (3C, 4H, BE3C, BF4T, BT, CASM3C, HDF3CGF, Mphg, KF3CT, LPS, MyoF, SAg) encompassing 148 total endpoints (Tab. S2^3^). For bioactivity testing, an active or positive hit call was made for each endpoint and the lowest effective concentrations determined for those considered active (Tab. S3^7^, see column “modl_ga”). Assay reproducibility was evaluated using the qualitative concordance of active hit calls for blinded sample replicates. Overall, concordance was 96.6 ± 3.3%.

Cytotoxicity was determined in the assay systems by endpoints measuring total protein levels by SRB staining for adherent cell types, or metabolic activity by Alamar Blue for suspension cells (Shah, et al., 2017). Although effects on single cell systems may be indicative of selective effects on cell-specific targets, chemicals inducing positive results in multiple cytotoxicity endpoints may reflect non-specific, general cell stress mechanisms. Here we considered samples active in two or more cytotoxicity-associated endpoints as nonspecifically cytotoxic. Those compounds, the number of cytotoxicity endpoints positive, and the active concentrations are shown in Table 1. A range of structural features was noted including carboxylic and sulfonic acids, sulfonamides, alcohols, and diacrylates. Cell type-selective cytotoxicity patterns were previously observed in testing other chemical classes (Houck et al., 2009).

**Fig. 1:**
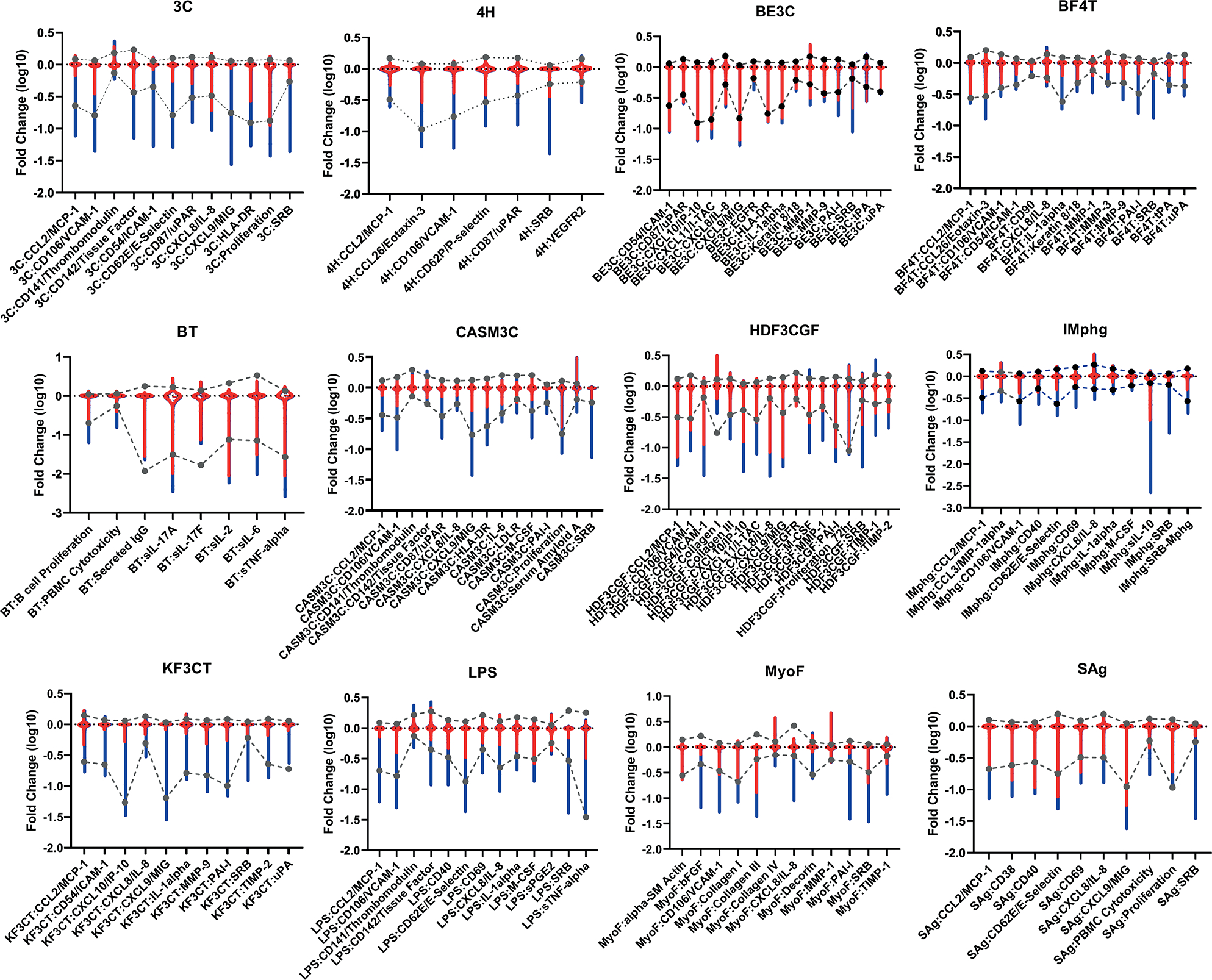
Range of responses for the 12 cell systems in the BioMap Diversity Plus panel of cell systems The fold change (log_10_) values for all PFAS samples at all concentrations for each assay endpoint are shown in red and grouped by cell system (cell system abbreviations are shown above each graph and detailed in the Materials and Methods). The range after removing data from chemical-concentration pairs where two or more cytotoxicity endpoints were active (blue) is indicated along with the 1–99% range for historic values after cytotoxicity filtering (dotted lines).

First, we wanted to understand if the log_10_-fold change and potency values observed for PFAS responses were similar to previous screenings with other chemicals. To achieve this, responses with PFAS samples were compared to previously collected data from a diverse collection of environmental chemicals to evaluate whether the PFAS, as a class of chemicals, showed distinctly different behavior in terms of efficacy (Kleinstreuer, et al., 2014). The range of responses in fold-change (log_10_) for all endpoints and all concentrations is shown in Figure 1. The full range (red) and the range after removing data from chemical-concentration pairs where two or more cytotoxicity endpoints were active (blue) are indicated along with the 1–99% range for historic values after cytotoxicity filtering (dotted lines) in these assays. The decreased range in the inhibitory direction shows that cytotoxicity had strong inhibitory effects on many endpoints. The PFAS responses in most cell systems, after cytotoxicity filtering, were within the ranges seen for previous environmental chemical testing (dotted lines).

**Fig. 2:**
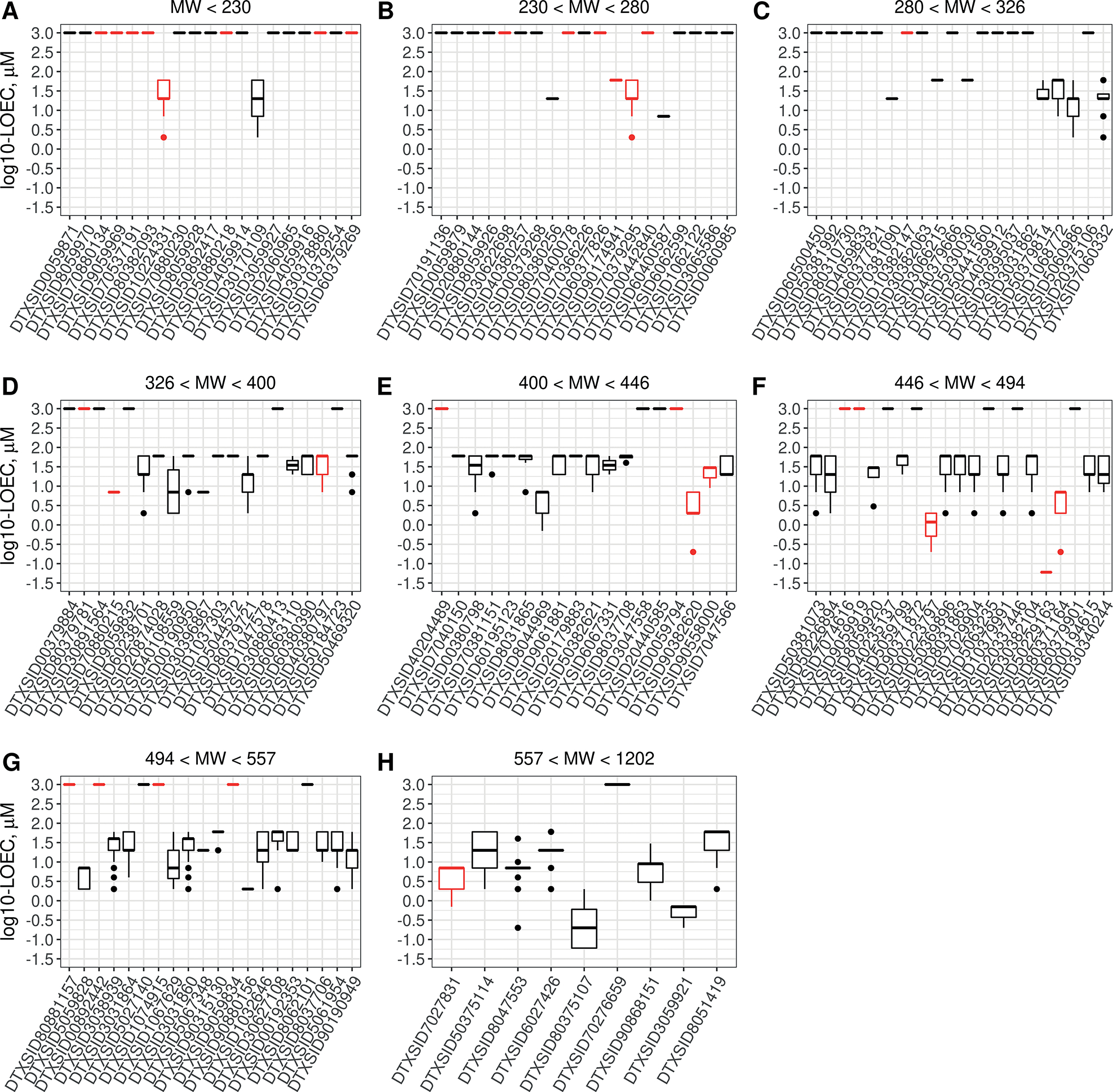
Potency of PFAS in BioMAP Boxplots indicate the median and interquartile range of lowest effect concentrations by PFAS for all endpoints, with the PFAS grouped by increasing molecular weight as indicated by the title label for each subplot, A-H. Chemicals are identified by DTXSID, and chemical name and analytical chemistry analysis results are available in Table S2^3^. Lines at 3.0 on the y-axis indicate PFAS that were negative in the BioMAP panel. Black = passing analytical QC; red = analytical QC denoted as failure.

Next, we wanted to understand the potencies of BioMAP responses for PFAS. The potency range of PFAS responses in all 12 assays is shown in Figure 2. PFAS are arranged from lowest molecular weight to highest and represented by their respective DSSTox substance identifiers, or DTXSIDs (Grulke et al., 2019). Lines at 3.0 on the y-axis (equivalent to 1000 μM and well above screened concentrations) in Figure 2 indicate that the PFAS sample had no bioactivity in any of the BioMAP assays screened. It is evident that lower molecular weight PFAS have much less activity than higher mass ones. There were also significantly more samples of lower molecular weight that failed analytical chemistry quality control, as shown by the red symbols. This may be due to increased likelihood of volatilization and loss of expected parent structure. Almost all of the bioactivity observed in the 12-assay BioMAP system was between approximately 3 and 60 μM, similar to the range of potencies observed for other chemicals that have been screened previously (Kleinstreuer et al., 2014).

### 3.3 Similarity to reference immunosuppressants using self-organizing maps

**Fig. 3:**
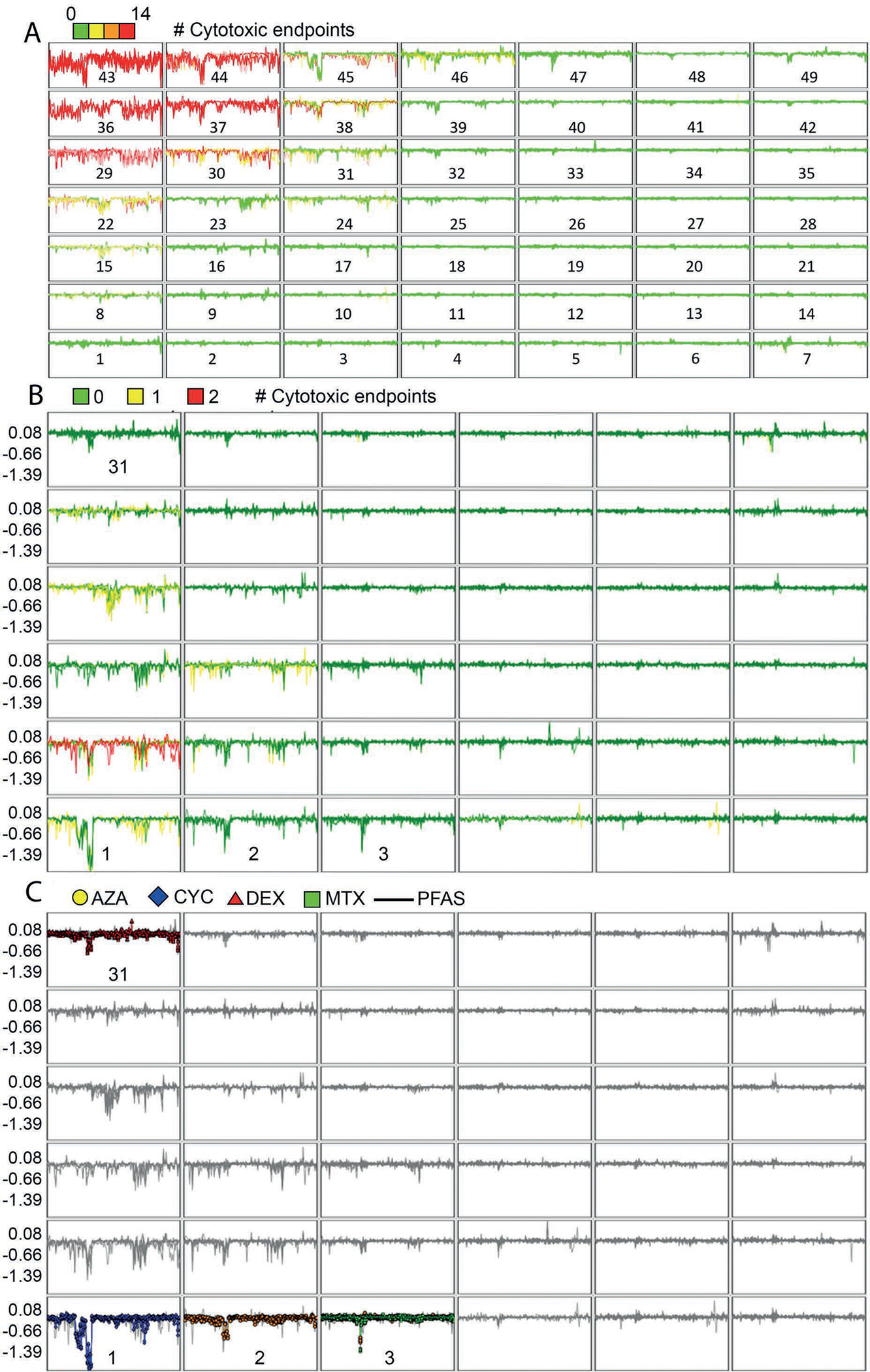
Response profile clustering by self-organizing maps (SOMs) (A) SOM for entire BioMAP panel result set. Chemical-concentration profiles at individual concentrations were clustered into a seven-by-seven array. Profiles were colored by the number of positive cytotoxicity endpoints for each chemical-concentration pair, with orange-red colors suggesting higher numbers of positive cytotoxicity endpoints. Clusters are numbered from lower left to top right. (B) SOM for BioMAP panel result set with cytotoxicity profiles removed. Individual chemical response profiles at each tested concentration, after removal of response profile with more than two positive cytotoxicity endpoints, were clustered again by the SOM method into a six-by-six array. Cluster numbers are from lower left to top right and selected cluster numbers are shown as they are discussed further in Results. The coloring in 3B indicates 0 (green), 1 (yellow) and 2 (red) cytotoxicity endpoints positive for chemicals in the cluster. (C) SOM for BioMAP panel results with immunosuppressive reference chemicals highlighted. Clusters are the same as in Figure 3B but with PFAS profiles shown in gray and reference immunosuppressant profiles in color as indicated in the legend (AZA, azathioprine in gold; CYC, cyclosporine A in blue; DEX, dexamethasone in brown; MTX, methotrexate in green).

Similarity between the bioactivity response profile of reference immunosuppressants and screened PFAS samples was examined using self-organizing maps (SOM). The hypothesis behind this comparison is that if PFAS bioactivity response profiles are similar to the response profiles of reference immunosuppressive drugs, then PFAS and these reference immunosuppressive drugs may act upon the same biological targets within the BioMAP system of assays. To examine the degree of similarity between PFAS and immunosuppressive drug responses, we clustered results across all endpoints at the individual testing concentration using SOM methodology. The 1208 response profiles (151 chemical samples/four concentrations/duplicate samples) were clustered in a 7X7 array that resulted in clusters strongly influenced by the cytotoxicity endpoints (Fig. 3A). In particular, clusters labeled 43, 44, 45, 36, 37, 38, 29, 30, 31, and 22 appear highly influenced by cytotoxicity, with higher numbers of positive cytotoxicity endpoints in those clusters. Clusters that lacked a high number of active cytotoxicity endpoints appeared to demonstrate little activity across the SOM panels (mostly flat green lines). To accommodate this bias towards overall suppression of endpoints secondary to general cytotoxicity, we removed concentration-response profiles for concentrations associated with greater than two positive cytotoxicity endpoints and reclustered using a 6X6 SOM array. This clustering is shown in Figure 3B with the presence of positive cytotoxicity endpoints shown by color. In Figure 3C, the profiles of the reference immunosuppressants are indicated by color as detailed in the legend. This resulted in the reference compounds being grouped into mostly distinct clusters. The exception was for azathioprine and methotrexate, which clustered similarly, both with mechanisms of action as antimetabolites inhibiting cell cycle S phase (Bertino, 1973). Full clustering results are provided in Table S4^8^. Additionally, in the paragraphs that follow, we discuss the chemicals included within the SOM clusters labeled 1, 2, 3, and 31 from Figures 3B–C in more detail.

**Fig. 4:**
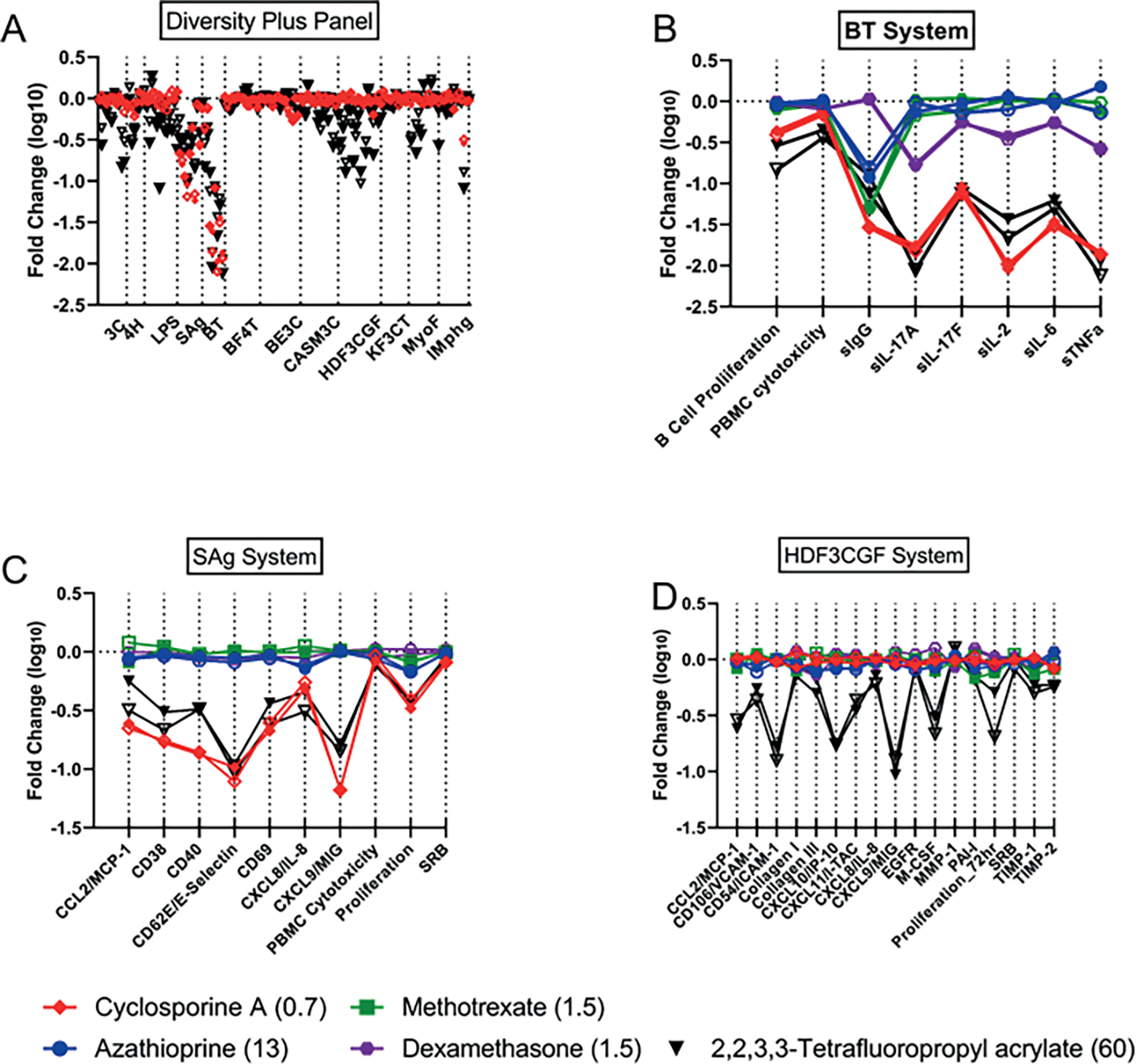
Comparison of cyclosporine A with 2,2,3,3-tetrafluoropropyl acrylate The Y-axes on 4A-D all represent the log_10_-fold change observed with a given chemical. Responses across all Diversity Plus panel endpoints for 2,2,3,3-tetrafluoropropyl acrylate (60 mM) (black) and cyclosporine A (0.67 mM) (red) are shown in (A) with the two replicates indicated by open or closed symbols. Responses for the individual cell systems BT (B), SAg (C), and HDF3CGF (D) show more detail and also include the reference immunosuppressants azathioprine (13 mM) (blue), methotrexate (1.5 mM) (green), and dexamethasone (1.5 mM) (purple). The X-axis labels in (B), (C), and (D) represent measurements made within those systems such as cytokines secreted or cellular proliferation.

All concentrations tested of the potent immunosuppressant cyclosporine A, except for the highest (18 mM), which was cytotoxic in five endpoints, were in SOM cluster #1. Effects were predominantly in the SAg and BT cell systems. A single PFAS, 2,2,3,3-tetrafluoropropyl acrylate, was also in cluster #1 at its highest tested concentration, 60 mM. A comparison of 2,2,3,3-tetrafluoropropyl acrylate and cyclosporine A is shown in Figure 4A. Suppression of multiple endpoints in the BT system (Fig. 4B) and the SAg system (Fig. 4C) is similar for both chemicals with strong reduction in secreted IgG and the cytokines IL-17A, IL-2, IL-6, and TNFα in the BT assay. Notably, while cyclosporine A was very selectively active for these two assay systems, 2,2,3,3-tetrafluoropropyl acrylate was also active in others, in particular the wound healing and inflammation (HDF3CGF) system model in the context of Th1 type inflammation (Fig. 4D). It should be noted that the analytical QC score for 2,2,3,3-tetrafluoropropyl acrylate indicated that some degradation of the parent structure was evident, thus confounding interpretation of the bioactivity testing. More specifically, though bioactivity was evident for the sample of 2,2,3,3-tetrafluoropropyl acrylate, it is unclear if the bioactivity observed was at the nominal concentration reported or if the observed bioactivity was related to a degradant in the sample.

**Fig. 5:**
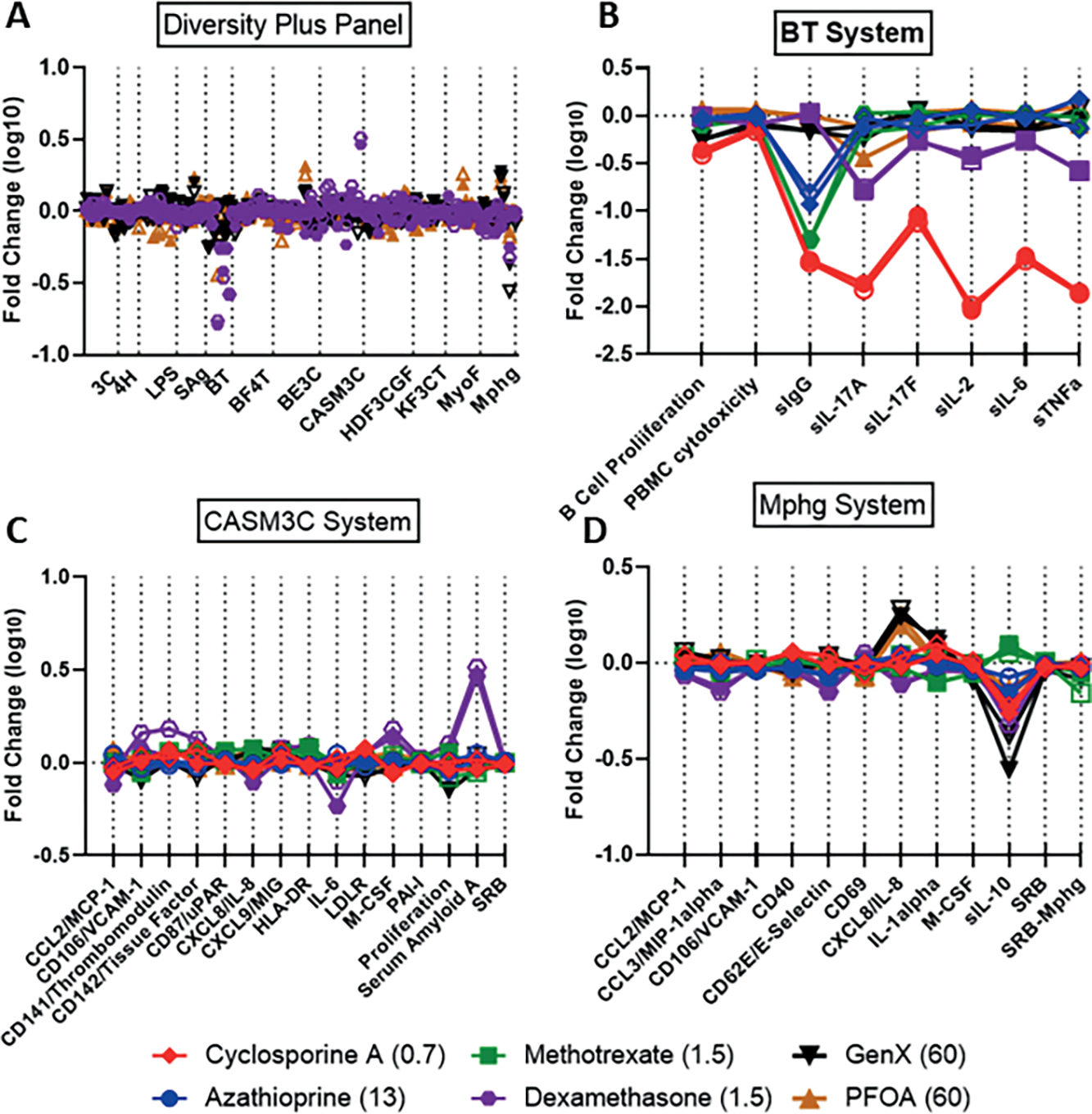
Comparison of dexamethasone with GenX and PFOA in the full Diversity Plus panel, BT, CASM3C, and Mphg systems The Y-axes on 5A-D all represent the log_10_-fold change observed with a given chemical. Responses across all Diversity Plus panel endpoints for GenX (60 mM) (black), PFOA (60 mM) (brown), and dexamethasone (1.5 mM) (purple) are shown in (A) with the two replicates indicated by open or closed symbols. Responses for the individual cell systems BT (B), CASM3C (C) and Mphg (D) show more detail and also include the reference immunosuppressants cyclosporine A (0.67 mM) (red), azathioprine (13 mM) (blue), methotrexate (1.5 mM) (green), and dexamethasone (1.5 mM) (purple).

Cluster #31 contained all tested concentrations of dexamethasone, a glucocorticoid causing immunosuppression through activation of the glucocorticoid receptor resulting in transrepression (repression of gene activation by other transcription factors) of many inflammatory cytokine genes (Stahn et al., 2007). There were nine different PFAS samples included in this cluster, all but one with both replicates at one or more concentrations tested. Ammonium perfluorooctanoate (two samples at 60 mM) and perfluorooctanoic acid (one sample at 60 mM), and both forms of PFOA, were present. Ammonium perfluoro-2-methyl-3-oxahexanoate, also known as GenX, was present in cluster #31 at all four concentrations tested and all replicates. The PFAS in cluster #31 have considerable structural similarity; all PFAS in this cluster contain linear perfluoroalkyl chains of medium length with carboxylic or sulfonic acid head groups, with the exception of 1H,1H,10H,10H-perfluorodecane-1,10-diol, which contains diol head groups (Fig. S1^3^). Two of the PFAS in cluster #31 also have ether linkages in the perfluoroalkyl chains: perfluoro(2-ethoxyethane)sulfonic acid and GenX. Figure 5 compares the dexamethasone response at 13 mM with GenX and ammonium perfluorooctanoic acid at 60 mM. Similar to GenX and PFOA, all chemicals in cluster #31 showed suppression of IL-10 in the Mphg system. Dexamethasone also showed strong suppression of multiple cytokines in the BT system (Fig. 5B), while other cluster members, as illustrated by GenX and PFOA, had more modest to no effects. Finally, the acute phase serum amyloid A (SAA) protein was strongly increased by dexamethasone in the CASM3C system (Fig. 5C), consistent with known activity of the glucocorticoids acting through the glucocorticoid receptor in aortic smooth muscle cells (Kumon et al., 2001). However, none of the PFAS showed such activity.

**Fig. 6:**
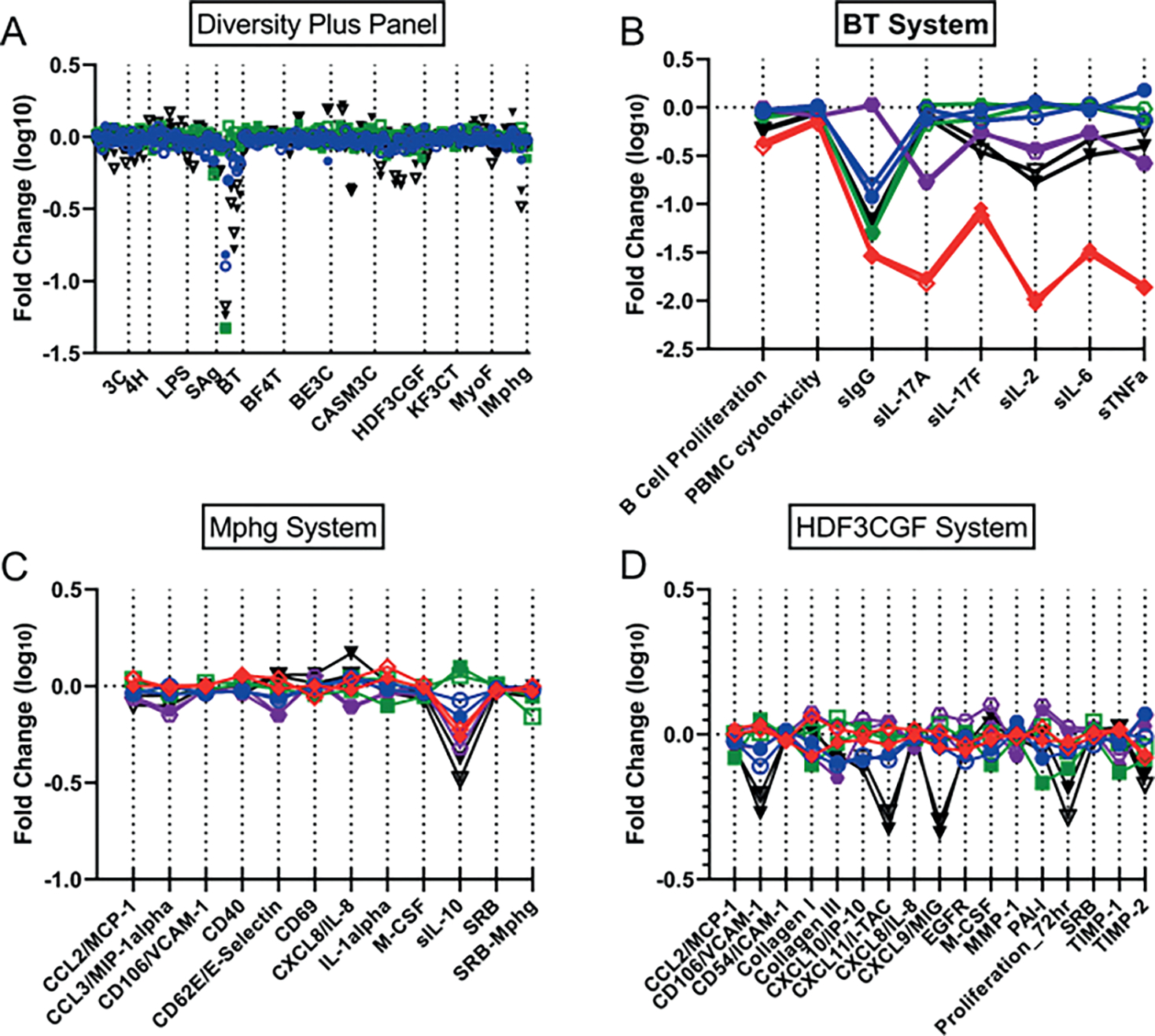
Similarity of 3H-perfluoro-2,2,4,4-tetrahydroxypentane with the reference immunosuppressants The Y-axes on 6A-D all represent the log_10_-fold change observed with a given chemical. Responses across all Diversity Plus panel endpoints for 3H-perfluoro-2,2,4,4-tetrahydroxypentane (60 mM) (black), azathioprine (13 mM) (blue), and methotrexate (1.5 mM) (green) are shown in (A) with the two replicates indicated by open or closed symbols. Responses for the individual cell systems BT (B), Mphg (C), and HDF3CGF (D) show more detail and also include the reference immunosuppressants cyclosporine A (0.67 mM) (red) and dexamethasone (1.5 mM) (purple).

The antimetabolites methotrexate and azathioprine clustered at all concentrations and replicates into cluster #2 of Figures 3B and 3C with the exception of the high concentration of azathioprine (38 mM), which fell into the adjacent (i.e., closely related) cluster #3. There were limited PFAS in clusters 2 and 3, and we consider both clusters together here. Three PFAS were present in clusters 2 and 3 as a single replicate at a single concentration, providing less confidence in their significance. 3,3-Bis(trifluoromethyl)-2-propenoic acid, 3H-perfluoro-2,2,4,4-tetrahydroxypentane, and perfluoropinacol were all included with both replicates at single concentrations (60, 60, and 20 mM, respectively). The most significantly modified endpoint for all chemicals in clusters 2 and 3 was reduction in secreted IgG in the BT cell system (Fig. 6). Reduced IgG is a reported effect *in vivo* for methotrexate and azathioprine when used for treatment of rheumatoid arthritis (Rackham et al., 2002; Kapetanovic et al., 2017; Levy et al., 1972). While methotrexate and azathioprine have minimal activity in other cell systems, the PFAS have a variety of additional activities including suppression of other cytokines in the BT cell system. In Figure 6, responses across all BioMAP Diversity Plus panel endpoints for the primary chemicals represented in clusters 2 and 3, including 3H-perfluoro-2,2,4,4-tetrahydroxypentane (60 mM) (black), azathioprine (13 mM) (blue), and methotrexate (1.5 mM) (green), are shown in (A), with replicates indicated by open and closed symbols. Responses for the individual cell systems BT (B), Mphg (C), and HDF3CGF (D) show more detail and include the reference immunosuppressants cyclosporine A (0.67 mM) (red) and dexamethasone (1.5 mM) (purple).

### 3.4 Bioactivity profiles as toxicity signatures

The profiles for PFAS chemicals were also analyzed for the presence of defined toxicity signatures from previous work with the BioMAP assay suite (Berg, 2019). These nine signatures are made up of 2–5 biomarker activities that have been associated with adverse effects and include acute toxicity, immunosuppression, skin irritation, liver toxicity, organ toxicity, skin rash, skin sensitization, thrombosis, and vascular toxicity. The immunosuppression signature tests for inhibition of B cell proliferation, T cell proliferation, cytotoxicity to PBMC, or inhibition of IgG secretion with all results at individual sample and concentration level are given in Table S5^9^. Thirty-one unique PFAS were flagged for the immunosuppression signature at one or more concentrations along with all four reference immunosuppressants (Tab. 2). Of these, 30 chemicals, including cyclosporine A, azathioprine, and methotrexate, were also flagged for the organ toxicity signature, which captures anti-proliferation effects in multiple cell types. Ten of the PFAS with immunosuppression signatures contained alcohol groups, six had acrylates, four carboxylic acids, and the rest had fewer common structural features. Also of note was the lack of any PFAS with a liver toxicity signature assigned for both sample replicates at any concentration although liver toxicity in animals has been associated with a number of PFAS including PFOA and PFOS (Lau et al., 2007). The BioMAP liver toxicity signature, however, only detects a vascular cell-driven specific steatosis-related mechanism of liver injury that is preferentially relevant to humans (Berg, 2019).

**Fig. 7:**
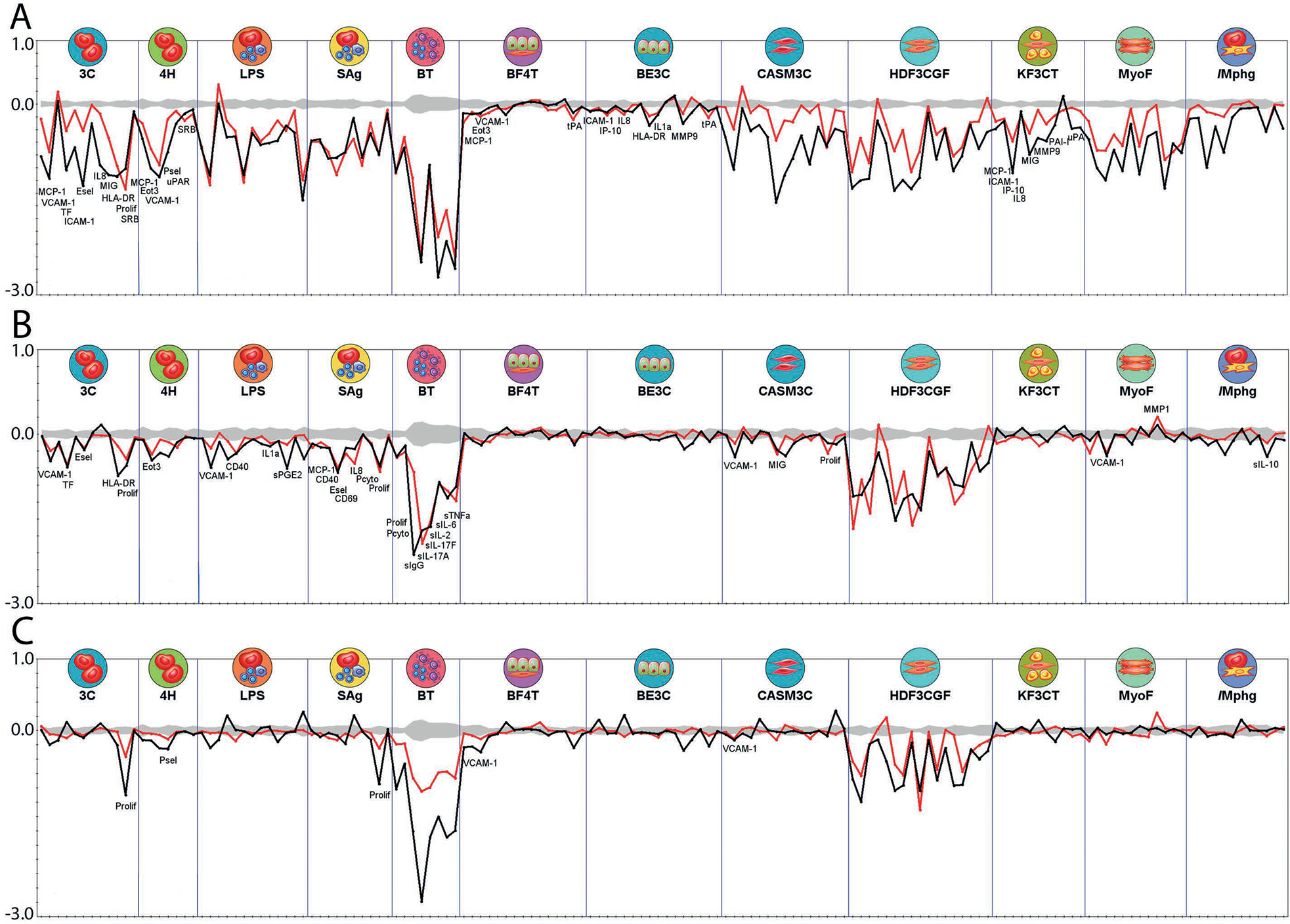
Comparison of response profiles in the 12 assay systems of the BioMAP Diversity Plus panel for PFAS with high Pearson’s correlations with reference pharmacological compounds from the BioMAP reference database Responses are shown as log_10_ fold-change over solvent control. Statistically significant responses extend beyond the gray shaded region. (A) 1H,1H,5H,5H-Perfluoro-1,5-pentanediol diacrylate (30 mM, red line) and the ubiquitin ligase inhibitor Ro 106–9920 (10 mM, black line). (B) 1H,1H,5H,5H-Perfluoro-1,5-pentanediol diacrylate (6.7 mM, red line) and the anti-angiogenic drug TNP-40 (10 mM, black line). (C) 1H,1H,6H,6H-Perfluorohexane-1,6-diol diacrylate (6.7 mM, red line) and the antibiotic auranofin (370 nM, black line).

**Fig. 8:**
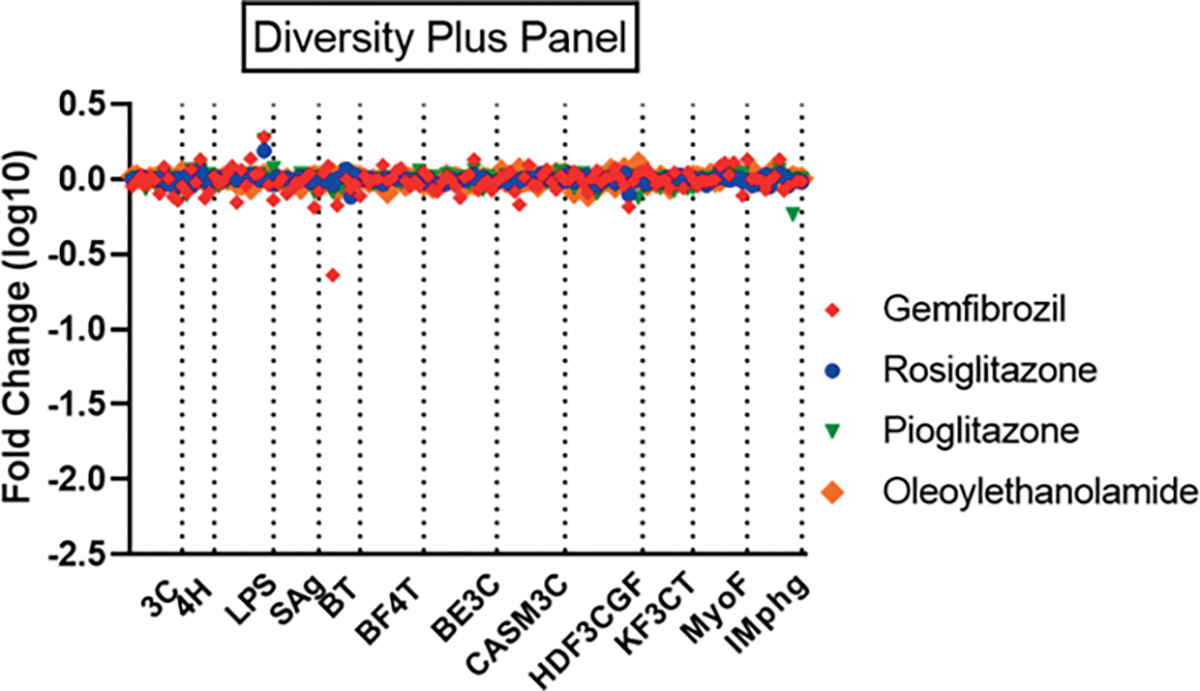
PPAR reference agonist effects in Diversity Plus panel Profiles for PPARg agonists rosiglitazone (3.7 μM, blue circle), pioglitazone (10 μM, green triangle) and PPARa agonists gemfibrozil (200 μM, red diamond) and oleoylethanolamide (1.1 μM, large orange diamond) are shown for the 12 assay systems of the BioMAP Diversity Plus panel. Concentrations were selected from the database to exceed reported *in vitro* EC50 values for the corresponding receptor targets by 5- to 40-fold.

PFAS activity profiles were also tested for similarity to the Eurofins Discovery database of reference profiles developed through screening diverse pharmacological and environmental chemicals. The purpose of this analysis was to identify hypotheses for additional mechanisms of action for the PFAS in this screen, many of which are data-poor, based on the similarity between their BioMAP response profiles to data from chemicals with known mechanisms of action and/or biological targets. The top ten matches with Pearson’s correlations greater than 0.6 for each PFAS are listed in Table S6^10^. The four reference immunosuppressants tested in this study were included in the evaluation and returned the corresponding matches from the database at one or more concentrations. One of the most striking similarities noted was between seven PFAS structures, many of which were structurally related, and inhibitors of the proteosome pathway (Tab. S7^10^). PFAS chemicals with both replicates at one or more concentrations having Pearson correlations above 0.7 matching the proteasome deubiquitinase inhibitor VLX1570 included four PFAS acrylates (1H,1H,5 H,5H-perfluoro-1,5-pentanediol diacrylate; 1H,1H,6H,6H-perfluorohexane-1,6-diol diacrylate; 2-(perfluorobutyl)ethyl acrylate; 2,2,3,3-tetrafluoropropyl acrylate) and 7:3 fluorotelomer alcohol; three of these (1H,1H,5 H,5H-perfluoro-1,5-pentanediol diacrylate; 1H,1H,6H,6H-perfluorohexane-1,6-diol diacrylate; 7:3 fluorotelomer alcohol) also matched the ubiquitin ligase inhibitor Ro 106–9920. Two PFAS, 3-(perfluorooctyl)propanol and 3,3-bis(trifluoromethyl)-2-propenoic acid, matched the profile of bortezomib, a proteasome inhibitor that binds to the catalytic site of the 26S proteasome (Bonvini et al., 2007). Proteasome inhibitors have been previously reported to be immunosuppressive (Khalesi et al., 2021). Other matches for the acrylates included the acetaldehyde dehydrogenase inhibitor disulfiram, the antibiotic auranofin, and the anti-angiogenic drug TNP-40. Figure 7 illustrates the similar results across all endpoints for two diacrylates, 1H,1H,5 H,5H-perfluoro-1,5-pentanediol diacrylate and 1H,1H,6H,6H-perfluorohexane-1,6-diol diacrylate with Ro 106–9920, TNP-40, and auranofin. Several PFAS, including perfluoroundecanoic acid, 11-H-perfluoroundecanoic acid, perfluorodecanoic acid, perfluoro-3,6,9-trioxatridecanoic acid, PFOS, and 9-chloro-perfluorononanoic acid, were similar to thyroid hormone (TH) or TH analogs with Pearson’s correlations greater than 0.7. However, the PFAS matched concentrations of the TH agents (micromolar) that were much higher than physiological concentrations, suggesting that this similarity may be due to non-TH targets. Only weakly supported matches to two known targets of several PFAS, the fatty acid-activated nuclear receptors PPARa or PPARg, were found. 1-Iodo-1H,1H,2H,2H-perfluorononane at two concentrations (but only single test samples) matched the endogenous PPARa agonist oleoylethanolamide and a single sample of perfluorohexanesulfonamide at 60 mM single matched that of rosiglitazone (33 mM). However, prior testing experience yielded limited responses to reference PPAR agonists at pharmacological concentrations, suggesting limited target expression/function in these cell systems. Profiles for PPARa agonists oleylethanolamide and gemfibrozil and PPARg agonists rosiglitazone and pioglitazone at concentrations exceeding their effective *in vitro* reported ones are shown in Figure 8 (Fu et al., 2003; De Filippis et al., 2015; Young et al., 1998; Sakamoto et al., 2000).

Finally, we provide a link to all fold-change results at the individual assay endpoint for each sample at each concentration tested in the form of a Tableau workbook^11^. This allows interactive exploration of the full PFAS data set. The breadth and complexity of the data limit the analysis that can be provided in this manuscript, and it is hoped that the access to these data will serve as a resource for hypothesis generation to the PFAS research community.

## 4 Discussion

Here we tested the effects of a diverse collection of 147 PFAS chemicals in a large panel of complex human primary cell systems and evaluated results to identify potential mechanisms of toxicity. This evaluation involved analysis of the bioactivity of PFAS in specific assays representing human pathophysiological states, as well as analysis of the similarity of the overall bioactivity response profile for all 12 assays between PFAS and chemicals with known molecular targets. As immunosuppression has been reported as an adverse effect of *in vivo* PFAS exposure, we compared the bioactivity response profiles of PFAS to four well-known immunosuppressants to specifically address potential mechanisms of immunotoxicity of PFAS. The association of PFOA and PFOS with immunotoxicity was previously concluded through a systematic review of human and animal studies as well as *in vitro*/mechanistic studies (NTP, 2016); PFOA and PFOS were “*presumed to be an immune hazard to humans based on a high level of evidence that PFOA suppressed the antibody response from animal studies and a moderate level of evidence from studies in humans*.” The European Food Safety Authority extensively reviewed both animal studies and human epidemiological associations of PFAS (primarily PFOA and PFOS) and reported decreased T cell-dependent antibody responses and reduced antibody response to vaccinations as critical indicators of PFAS effects (EFSA, 2020). Our results provide only limited mechanistic support for these conclusions for PFOA and PFOS. PFOA and GenX appeared to suppress IL-10 in the Mphg system, like dexamethasone; unlike dexamethasone and the other immunosuppressive drugs in this work, PFOA and GenX had modest to no effects on suppression of cytokine production in the BT system. However, several other PFAS, i.e., 3-bis(trifluoromethyl)-2-propenoic acid, 3H-perfluoro-2,2,4,4-tetrahydroxypentane, and perfluoropinacol, have activities more similar to the reference immunosuppressants in some of the cell systems, including suppression of IgG secretion. Importantly, it should be noted that our analysis relied principally on correlation of bioactivity signatures with pharmacological mediators of immunosuppression with specific mechanisms of action; other potential pathways or more non-specific and pleiotropic modes of action resulting in immune suppression by PFAS such as PFOA and/or PFOS that may not be captured by the BioMAP panel cannot be ruled out. For example, systemic toxicity and stress in the whole animal has been postulated as a mechanism of multiple immune suppression effects for PFOS and PFOA, and these are modes-of-action that may not be captured in the BioMAP panel (Loveless et al., 2008). Other studies have suggested that alterations in IgG secretion in mice exposed to PFOA may occur independently of systemic toxicity and stress-related corticosterone increases (DeWitt et al., 2009a), noting that IgG secretion in the BT system in BioMAP was unaffected by PFOA. Future studies measuring the effects of environmental chemicals associated with immunotoxicity in the BioMAP co-culture systems, along with other human cell-based models of immune related effects including inflammation, may be useful for better defining the bioactivity profiles of non-pharmaceutical immunotoxic compounds and understanding mechanisms of putative immune related effects in human populations.

Beyond immunosuppression, significant bioactivity in human primary cells that correlated with mechanisms of action that may indicate potential for adverse effects *in vivo* was observed for diverse PFAS as discussed below. From the BioMAP Diversity Plus co-culture systems, the BT assay seemed most appropriate to directly examine the association of PFOA, PFOS, and other PFAS with effects on B cell antibody responses. The BT cell system, consisting of co-cultures of B cells and peripheral blood mononuclear cells (PBMCs) and stimulated with anti-IgM and low levels of TCR ligands over three days (soluble endpoints) or six days (IgG) of chemical exposure, was highly responsive to all the reference immunosuppressants (see also Melton, 2013). Distinct responses between the three mechanisms of action of these reference compounds were readily seen and likely drove their dissimilar clustering. Notably, both the antimetabolites (azathioprine and methotrexate) and cyclosporine A had strong suppression of IgG secretion in the stimulated B cells, while dexamethasone did not suppress IgG but did have strong effects on cytokine secretion endpoints. Based on the NTP monograph (NTP, 2016), suppression of IgG might be expected for both PFOA and PFOS. However, neither had any effect on this endpoint. The NTP monograph did not include supporting *in vitro*/mechanistic data for IgG suppression but did report no suppression of IgM in either murine or human B cell lines, even at very high (750 mM) concentrations of PFOA (Levitt and Liss, 1986). It should be noted that these results were from cell lines constitutively secreting IgM and may not recapitulate the complexity of *in vivo* IgM secretion in response to an antigen stimulus. No results for effects of PFOS on B cell antibody production *in vitro* were identified in the review. Overall, neither PFOA nor PFOS seemed to show activity similar to the reference immunosuppressants except perhaps PFOA at its highest testing concentration of 60 mM where it clustered with dexamethasone in the SOM analysis. The association seemed relatively weak biologically as many of the cytokines modulated by dexamethasone were not affected by PFOA. In addition, with the lack of the characteristic increased SAA secretion as seen with dexamethasone, it seems unlikely the PFAS effect is directly through the glucocorticoid receptor. However, both PFOA and PFOS decreased the level of IL-10 in a co-culture system (Mphg assay) that detects macrophage responses. IL-10 is a cytokine that promotes B cell IgG production (Facciotti et al., 2020; Itoh et al., 1994). Whether this finding is relevant to immune responses in humans requires further study.

Comparison of PFAS response profiles to profiles of reference compounds within the BioMAP reference database containing pharmacological probes, drugs, and environmental chemicals identified a variety of relatively highly correlated matches to compounds with known mechanisms of action. The six PFAS acrylates were a particularly interesting example of apparent structural features driving biological activity. Profiles for five of the six acrylates showed high correlation with profiles from compounds that inhibit ubiquitin ligases involved in proteasome function. Proteasome inhibitors are used therapeutically as anticancer agents and drive cancer cells into apoptosis at appropriate concentrations (Kisselev and Goldberg, 2001). 1H,1H,6H,6H-Perfluorohexane-1,6-diol diacrylate showed responses similar to the ubiquitin ligase inhibitors VLX1570 (at 60 mM) and Ro 106–9920 (at 20 mM) and to the alcohol dehydrogenase inhibitor disulfiram at 2.2 and 6.6 mM. Disulfiram has recently been reported as having anti-cancer activity that may partly be explained by its ability to induce autophagy with the latter process known to be stimulated by inhibition of proteasome activity (Ji and Kwon, 2017). Further, 1H,1H,9H-perfluorononyl acrylate and 1H,1H-perfluorooctyl acrylate showed similarity to auranofin, an antibiotic that can inhibit thioredoxin reductase, an enzyme that maintains cellular redox potential and whose expression can be repressed by the proteasome inhibitors bortezomib and carfilzomib (Fink et al., 2016). The other major correlated profile was that of the fumagillin analog TNP-40, which inhibits angiogenesis through irreversible inactivation of methionine aminopeptidase-2 (MetAP2), blocking endothelial cell proliferation *in vitro* and angiogenesis *in vivo* (Griffith et al., 1998). MetAP2 cleaves the NH2-terminal methionine during nascent protein translation. Here, too, crosstalk with the proteasome pathway has been shown through TXNL1, a thioreductase that plays a role in the transfer of misfolded nascent protein chain from the ribosome to the 26S proteasome, which was unprocessed upon MetAP2 inhibition (Andersen et al., 2009). While proteasome inhibitors can be used therapeutically under controlled conditions, long-term exposure is toxic to nearly all cells by inducing apoptotic cell death (Kisselev and Goldberg, 2001). Side effects of proteasome inhibitors reported in clinical trials include anemia, gastrointestinal disorders, and peripheral neuropathy (Hungria et al., 2019).

Cyclosporine A had selective cell system activity at three of four concentrations tested with activity primarily in the SAg and BT systems. Presumably, this activity results from the known mechanism of action for cyclosporin A: complexing with cyclophilin and subsequent inhibition of the phosphatase activity of calcineurin preventing nuclear translocation and activation of NFAT transcription factors (Matsuda and Koyasu, 2000). In addition to the calcineurin/NFAT pathway, recent studies indicate that cyclosporin A also blocks the activation of JNK and p38 signaling pathways triggered by antigen recognition, making cyclosporin A a highly specific inhibitor of T cell activation. At 18 mM, however, cyclosporin A affected additional cell systems with significant suppression of a variety of endpoints. Similarity analysis at this concentration indicated a high correlation of the profile with that of omipalisib, a phosphoinositide 3-kinase inhibitor that blocks activity of the mammalian target of rapamycin complex 1 (mTORC1) (Knight et al., 2010). Such activity may be consistent with reported anticancer effects of cyclosporin A at micromolar concentrations, with activity attributed to inhibition of mTORC1 signaling in prostate cancer cells (Lee et al., 2012). Note that this concentration is greater than typical pharmacological exposures in patients treated with cyclosporin A as immunosuppressant therapy (with a maximum plasma concentration, Cmax, of < 1–2 mM) (Halloran et al., 1999). Profiles of 8:2 fluorotelomer alcohol at 60 mM (both replicates) and N-ethyl-N-(2-hydroxyethyl)perfluorooctanesulfonamide at 60 mM (single replicate) also had relatively high similarity with the profile of omipalisib, supporting involvement with mTORC1 activity for high concentrations of these specific PFAS.

The primary conclusion from our work was a finding of very limited similarity of PFOA and PFOS to the bioactivity profiles of reference immunosuppressants that exhibit three important clinical mechanisms of action. The human primary cell systems in the BioMAP suite responded robustly in relevant endpoints to the reference compounds, supporting the conclusion that these assays are sensitive to these immunosuppressive mechanisms. While several PFAS had activity profiles that overlapped with the reference compounds, those PFAS had additional activities that confound interpretation of potential immunosuppressive-specific effects. These results should inform future studies where potential exposure scenarios indicate a possibility of risk. A limitation of this work is that not all potential mechanisms of immunosuppression are functional in the assay systems used here. For instance, PPARa and PPARg are known molecular targets for PFAS, and among numerous bioactivities associated with these receptors in the literature are effects on the immune system, primarily categorized as anti-inflammatory and suggested as a mechanism for PFAS immunosuppression (Christofides et al., 2021; Peden-Adams et al., 2008). The PPARs primarily regulate metabolism in many cell types, which impacts the differentiation, expansion, and fate commitment of immune cells (Christofides et al., 2021). Some of these critical steps in development of a fully functional immune system are not present in these relatively short-term *in vitro* assays and, thus, these assays may not detect putative contributions of PPAR signaling pathways to immune function. PPARs are also known to function in large complexes that include various co-activators and co-repressors that impart cell-type and tissue-specific effects, with considerable species differences including in immune responses (Yu and Reddy, 2007; Corsini et al., 2014). Thus, there is uncertainty about the role of PPARs in any potential immune effects of PFAS in humans *in vivo*, based on lower expression of PPARa in humans when compared to rodents as well as data from mouse models that suggests that some immune-related effects observed in mice following PFOA and PFOS exposure may be independent of PPARa (DeWitt et al., 2009b).

Additionally, PPARa agonists oleylethanolamide and gemfibrozil and PPARg agonists rosiglitazone and pioglitazone failed to produce many effects in the BioMAP suite at the concentrations screened. A lack of PPAR responsiveness in the 12-assay BioMAP assay suite and absence of the liver toxicity signature for these PPAR agonists is of interest given that PPAR-like liver effects have been shown *in vivo* for a number of PFAS including PFOA and PFOS (Perkins et al., 2004; Bjork et al., 2008; Bjork and Wallace, 2009). This is not necessarily surprising given that the BioMAP-defined liver toxicity signature used for this work is a specific mechanism-based signature that relates to steatosis liver injury in humans involving vascular cells (Berg, 2019). It does not detect other mechanisms of liver injury. It is postulated that the liver effects seen in rodent studies with PFOA and PFOS are PPAR-mediated and, assuming limited PPAR sensitivity of the cell systems used here, the BioMAP assays would be unlikely to be capable of capturing PPAR-mediated liver effects. Further, the BioMAP assays used human primary cells; even if these systems were responsive to PPAR agonists, the PPARa-linked liver toxicity for PFAS is argued as not relevant to human exposure (Corton et al., 2018).

The testing concentrations selected for this study were determined based on the ability to generate a universal solubilization method at relatively high concentration in DMSO for a diverse PFAS library. *In vitro* testing concentrations were then determined by the 0.1% limit of DMSO tolerability of these assay systems. Thus, the concentration series for the PFAS were generally from 2–60 mM (Tab. S1^1^; some lower due to solubility limitations). These concentrations exceed by two orders of magnitude the serum levels measured in the 2015–16 NHANES study, which reported geometric means of 3.8 nM PFOA and 9.4 nM PFOS for the general US population (CDC, 2019). However, human exposures can be considerably higher as, for example, those seen in the population exposed to heavily contaminated drinking water from industrial processes in West Virginia where geometric mean PFOA concentration was 193 nM with extremes up to 42 mM and mean PFOS of 44 nM ranging up to 1.5 mM (Steenland et al., 2009). An additional factor that is likely to be important for extrapolating *in vitro* activity to *in vivo* effects is the actual exposure levels of PFAS to the cells in the testing system. Here we used nominal cell culture medium concentrations; however, there are numerous parameters affecting actual exposure that need consideration (Armitage et al., 2014; Wambaugh et al., 2019). Indeed, PFAS have been shown to have significant effects on several properties including serum protein binding and bioaccumulation that may impact actual cellular exposure levels (Yang, D. et al., 2020; Zhang, W. et al., 2020).

An additional important consideration of the approach used for this study is the reliance on primary cells from a limited number of donors. Donor cell pooling provides strength in not relying solely on the genotype and phenotype of a single individual that may bias results. It also ensures a larger supply of cells needed to determine levels of experimental variability. However, it does not guarantee that any potentially more susceptible donor subpopulations are included in the pool or whether such phenotypes would not be more difficult to detect as only a small part of a broader pool of donors. Sex-differences, age-differences, and race/genetic differences in immune cells can influence the outcome of the bioassays (Scepanovic et al., 2018); however, the magnitude of increased testing that would be required to evaluate this is beyond the resource scope of this project.

We have provided an overview of the effects of a diverse set of PFAS, many with known human exposures and measured blood levels, in human primary cells and identified numerous perturbations of important cellular signaling and response systems. While we have focused on a limited number of specific effects, including similarity to activity of clinical immunosuppressant drugs, the data set likely contains many additional activities of interest. We provide access to these data through a publicly accessible database that allows interactive exploration of the full data set to aid in hypothesis generation for PFAS mechanisms of action^11^. These data will also be used to help develop a categorization system for PFAS combining chemical structural information with bioactivity data (Patlewicz et al., 2019). While no detailed analysis of structure-function correlation was performed for this analysis, suggestions of such associations were seen such as for the links to the proteasome pathway by PFAS-containing acrylate substructures. Categorization may be one important tool to aid in prioritizing PFAS for safety assessment as well as contribute to risk analysis in facilitating grouping of PFAS from complex mixtures that may have additive or other interactive activities (Ojo et al., 2020).

## Supplementary Material

Supplement1

Supplement2

Supplement3

Supplement4

Supplement5

Supplement6

## Figures and Tables

**Tab. 1:** PFAS samples with cytotoxic activity PFAS samples that were associated with cytotoxicity and the average number of cytotoxicity endpoints active per chemical are indicated. The micromolar concentration of cytotoxic activity is provided, and an asterisk indicates where this was the highest concentration tested.

DTXSID	Preferred name	Concentrations active (*μ*M) (average of # cytotoxicity endpoints active)
DTXSID7060332	(Perfluorobutyryl)-2-thenoylmethane	60* (9)
DTXSID90190949	1,6-Diiodoperfluorohexane	60* (14); 20 (4.5)
DTXSID5061954	11-H-Perfluoroundecanoic acid	60* (4)
DTXSID50369896	1H,1H,10H,10H-Perfluorodecane-1,10-diol	60* (3)
DTXSID5060986	1H,1H,5H,5H-Perfluoro-1,5-pentanediol diacrylate	60* (9.5); 20 (3)
DTXSID80379721	1H,1H,6H,6H-Perfluorohexane-1,6-diol diacrylate	60* (8.5); 20 (3)
DTXSID40380797	1H,1H-Perfluoro-3,6,9-trioxadecan-1-ol	60* (4)
DTXSID10379991	3-(Perfluorooctyl)propanol	60* (14); 20 (6.5)
DTXSID5044572	6:2 Fluorotelomer alcohol	60* (2.5)
DTXSID50382621	7:3 Fluorotelomer alcohol	60* (13); 20 (2)
DTXSID7029904	8:2 Fluorotelomer alcohol	60* (13.5); 20 (8)
DTXSID30382104	9-Chloro-perfluorononanoic acid	60* (4.5)
DTXSID0020365	Cyclosporin A	18* (5)
DTXSID6027426	N-Ethyl-N-(2-hydroxyethyl)perfluorooctanesulfonamide	60* (4.5)
DTXSID1032646	N-Ethylperfluorooctanesulfonamide	60* (5.5)
DTXSID80371164	Perfluoro(2-(2-propoxypropoxy)-1H,1H-propan-1-ol)	20* (3)
DTXSID50375114	Perfluoro-3,6,9-trioxatridecanoic acid	60* (8.5); 20 (5)
DTXSID3031860	Perfluorodecanoic acid	60* (11)
DTXSID8031863	Perfluorononanoic acid	60* (2.5)
DTXSID70381151	Perfluorooctanamidine	60* (3)
DTXSID3038939	Perfluorooctanesulfonamide	60* (11)
DTXSID8051419	Perfluorooctanesulfonamido ammonium iodide	60* (2)
DTXSID60238701	Perfluoropinacol	60* (7)
DTXSID8047553	Perfluoroundecanoic acid	20* (3)

* highest concentration tested

**Tab. 2:** Immunosuppressive toxicity signatures The toxicity signature for each chemical (if more than one sample concentration available, the minimum sample concentration associated with the signature is used) is summarized by the minimum concentration associated with the signature in micromolar concentration units or NA if the signature was not indicated. 31 PFAS and 4 reference immunosuppressants demonstrated the immunosuppression signature. DTXSID, DSSTox Substance Identifier; QC, summarization of analytical QC results for the chemical.

	DTXSID	Chemical name	Molecular weight (g/mol)	QC	Acute toxicity	Immunosuppression	Liver toxicity	Organ toxicity	Skin irritation	Skin rash	Skin sensitization	Thrombosis	Vascular toxicity
1	DTXSID80375107	11:1 Fluorotelomer alcohol	600.118	P	NA	0.4	NA	0.4	0.4	NA	NA	NA	NA
2	DTXSID0020365	Cyclosporin A	1202.635	N/A	18	0.7	NA	0.7	NA	NA	6	NA	NA
3	DTXSID3047429	Dexamethasone sodium phosphate	516.41	N/A	NA	1.5	NA	NA	NA	NA	1.5	NA	1.5
4	DTXSID4020822	Methotrexate	454.447	N/A	NA	1.5	NA	40	NA	NA	NA	NA	NA
5	DTXSID5060986	1H,1H,5H,5H-Per- fluoro-1,5-pentanediol diacrylate	320.187	P	60	2.2	NA	2.2	NA	NA	20	NA	NA
6	DTXSID80379721	1H,1H,6H,6H-Per-fluorohexane-1,6-diol diacrylate	370.195	P	60	2.2	NA	2.2	NA	NA	NA	NA	NA
7	DTXSID1 0224331	2,2,3,3-Tetrafluoro-propyl acrylate	186.106	P	NA	2.2	NA	2.2	NA	NA	2.2	NA	NA
8	DTXSID7060332	(Perfluorobutyryl)-2-thenoylmethane	322.2	P	60	2.2	NA	6.7	NA	NA	20	NA	NA
9	DTXSID40108559	Ammonium per-fluoro-2-methyl-3-oxa-hexanoate	347.084	N/A	NA	2.2	NA	2.2	2.2	NA	NA	2.2	NA
10	DTXSID4020119	Azathioprine	277.26	N/A	NA	4.2	NA	13	NA	NA	4.2	NA	NA
11	DTXSID40380797	1H,1H-Perfluoro-3,6,9-trioxadecan-1-ol	398.076	F	60	6.7	NA	20	NA	NA	NA	NA	NA
12	DTXSID00194615	1H,1H,9H-Perfluoro-nonyl acrylate	486.152	P	NA	6.7	NA	6.7	NA	NA	2.2	NA	NA
13	DTXSID50382621	7:3 Fluorotelomer alcohol	428.141	P	60	6.7	NA	6.7	NA	NA	NA	NA	NA
14	DTXSID6027426	2-Perfluorooctyl-sulfonyl-N-ethylamino-ethyl alcohol	571.25	P	60	6.7	60	2.2	NA	NA	20	6.7	6.7
15	DTXSID80371164	Perfluoro(2-(2-propo xypropoxy)-1H, 1H-propan-1-ol)	482.093	F	20	6.7	NA	6.7	NA	NA	NA	NA	NA
16	DTXSID90190949	1,6-Diiodoperfluoro-hexane	553.856	P	20	20	NA	6.7	NA	NA	NA	NA	NA
17	DTXSID5059799	1H,1H-Perfluorooctyl acrylate	454.135	P	NA	20	NA	20	NA	NA	NA	NA	NA
18	DTXSID1068772	2-(Perfluorobutyl)ethyl acrylate	318.139	P	NA	20	NA	6.7	NA	NA	60	NA	NA
19	DTXSID20874028	2H,2H,3H,3H-Per-fluorooctanoic acid	342.108	P	NA	20	NA	NA	NA	2.2	6.7	NA	NA
20	DTXSID30170109	3,3-Bis(trifluoromethyl)-2-propenoic acid	208.059	P	NA	20	NA	20	NA	NA	6.7	NA	NA
21	DTXSID70379295	3H-Perfluoro-2,2,4,4-tetrahydroxypentane	262.08	F	NA	20	NA	20	60	NA	20	NA	60
22	DTXSID1067629	N-Methylperfluoro-octanesulfonamide	513.17	P	NA	20	NA	20	NA	NA	6.7	NA	60
23	DTXSID60400587	Nonafluoropentana-mide	263.063	P	NA	20	NA	NA	NA	NA	NA	NA	NA
24	DTXSID60238701	Perfluoropinacol	334.061	ND	60	20	NA	6.7	6.7	NA	NA	NA	NA
25	DTXSID9061881	1-Iodo-1H,1H,2H,2H-perfluoroheptane	423.996	P	NA	60	NA	20	NA	NA	NA	NA	NA
26	DTXSID50184723	1H,1H-Perfluoro-octylamine	399.103	ND	NA	60	NA	NA	NA	NA	NA	NA	NA
27	DTXSID1047578	1H,1H,2H,2H-Per-fluorohexyl iodide	373.988	P	NA	60	NA	NA	NA	NA	NA	NA	60
28	DTXSID50369896	1H,1H,10H,10H-Per-fluorodecane-1,10-diol	462.13	P	60	60	NA	2.2	NA	NA	20	NA	NA
29	DTXSID00380798	1H,1H,11H,11H-Per-fluorotetraethylene glycol	410.112	P	NA	60	NA	2.2	20	NA	20	NA	NA
30	DTXSID00190950	6:1 Fluorotelomer alcohol	350.079	P	NA	60	60	60	NA	NA	60	NA	NA
31	DTXSID5044572	3,3,4,4,5,5,6,6,7,7,8,8, 8-Tridecafluorooctanol	364.106	P	NA	60	60	20	60	NA	NA	NA	NA
32	DTXSID3066215	(Heptafluorobutanoyl) pivaloylmethane	296.185	P	NA	60	NA	20	60	NA	2.2	NA	NA
33	DTXSID8031863	Perfluorononanoic acid	464.078	P	60	60	60	NA	NA	NA	2.2	NA	NA
34	DTXSID70381151	Perfluorooctanamidine	412.102	P	60	60	NA	20	NA	NA	20	NA	NA
35	DTXSID8051419	Perfluorooctanesulfonamido ammonium iodide	726.23	P	NA	60	NA	60	NA	NA	6.7	60	NA

## Data Availability

All supplementary files containing data described in this manuscript are available for download both at doi:10.23645/epacomptox.17131652 and at doi:10.14573/altex.2203041. These include: Table S1^1^: Quality control details on PFAS stock solution evaluations; Fig. S1^3^: Structures of chemicals in cluster #31; Table S2^3^: Endpoints evaluated; Table S3^7^: Complete bioactivity results from the ToxCast Pipeline, including the lowest effect concentration; Table S4^8^: SOM clustering results, including all concentrations of PFAS screened; Table S5^9^: Toxicity signature analysis, including all concentrations of PFAS screened; Table S6^10^: Chemical bioactivity similarity search results; Table S7^10^: Acrylates and proteasome pathway modulator similarity results.

## Footnotes

1 doi:10.14573/altex.2203041s1

2
https://comptox.epa.gov/dashboard/chemical_lists/epapfasinv

3 doi:10.14573/altex.2203041s2

5
https://comptox.epa.gov/dashboard/

6
https://CRAN.R-project.org/package=tcpl

7 doi:10.14573/altex.2203041s3

8 doi:10.14573/altex.2203041s4

9 doi:10.14573/altex.2203041s5

10 doi:10.14573/altex.2203041s6

11 https://public.tableau.com/app/profile/ellen.berg/viz/HouckEvaluationof147 PFASSubstances2022/CoverPage
