# Supplementary material for: Evaluation of 147 Perfluoroalkyl Substances for Immunotoxic and Other (Patho)physiological Activities through Phenotypic Screening of Human Primary Cells
Source: ALTEX. Author manuscript; Available in PMC 2024 Jan 1. (PMC10331698; doi:10.14573/altex.2203041)
Supplement: Supplement3 [file NIHMS1895629-supplement-Supplement3.pdf]

Houck et al.:

Evaluation of 147 Perfluoroalkyl Substances for Immunotoxic and Other (Patho)physiological Activities through Phenotypic Screening of Human Primary Cells

Supplementary Data

Tab. S2: Endpoints evaluated

System	Icon	Cell Type	Disease Relevance	Biomarker Readouts	Description
**3C**		Venular endothelial cells	Cardiovascular Disease, Chronic Inflammation	MCP-1, VCAM-1, TM, TF, ICAM-1, E-selectin, uPAR, IL-8, MIG, HLA-DR, Proliferation, SRB	The 3C system models vascular inflammation of the Th1 type, an environment that promotes monocyte and T cell adhesion and recruitment and is anti-angiogenic. This system is relevant for chronic inflammatory diseases, vascular inflammation and restenosis.
**4H**		Venular endothelial cells	Asthma, Allergy, Autoimmunity	MCP-1, Eotaxin-3, VCAM-1, P-selectin, uPAR, SRB, VEGFRII	The 4H system models vascular inflammation of the Th2 type, an environment that promotes mast cell, basophil, eosinophil, T and B cell recruitment and is pro-angiogenic. This system is relevant for diseases where Th2-type inflammatory conditions play a role such as allergy, asthma, and ulcerative colitis.
**LPS**		Peripheral blood mononuclear cells + Venular endothelial cells	Cardiovascular Disease, Chronic Inflammation	MCP-1, VCAM-1, TM, TF, CD40, E-selectin, CD69, IL-8, IL-1α, M-CSF, $sPGE_2$, SRB, sTNFα	The LPS system models chronic inflammation of the Th1 type and monocyte activation responses. This system is relevant to inflammatory conditions where monocytes play a key role including atherosclerosis, restenosis, rheumatoid arthritis, metabolic disease and other chronic inflammatory conditions.
**SAg**		Peripheral blood mononuclear cells + Venular endothelial cells	Autoimmune Disease, Chronic Inflammation	MCP-1, CD38, CD40, E-selectin, CD69, IL-8, MIG, PBMC Cytotoxicity, Proliferation, SRB	The SAg system models chronic inflammation of the Th1 type and T cell effector responses to TCR signaling with costimulation. This system is relevant to inflammatory conditions where T cells play a key role including organ transplantation, rheumatoid arthritis, psoriasis, Crohn's disease and multiple sclerosis.
**BT**		Peripheral blood mononuclear cells + B cells	Asthma, Allergy, Oncology, Autoimmunity	B cell Proliferation, PBMC Cytotoxicity, Secreted IgG, sIL-17A, sIL-17F, sIL-2, sIL-6, sTNFα	The BT system models T cell dependent B cell activation and class switching as would occur in a germinal center. This system is relevant for diseases and conditions where B cell activation and antibody production are relevant. These include autoimmune disease, oncology, asthma and allergy.
**BF4T**		Bronchial epithelial cells + Dermal fibroblasts	Asthma, Allergy, Fibrosis, Lung Inflammation	MCP-1, Eotaxin-3, VCAM-1, ICAM-1, CD90, IL-8, IL-1α, Keratin 8/18, MMP-1, MMP-3, MMP-9, PAI-1, SRB, tPA, uPA	The BF4T system models lung inflammation of the Th2 type, an environment that promotes the recruitment of eosinophils, mast cells and basophils as well as effector memory T cells. This system is relevant for allergy and asthma, pulmonary fibrosis, as well as COPD exacerbations.
**BE3C**		Bronchial epithelial cells	Lung Inflammation, COPD	ICAM-1, uPAR, IP-10, I-TAC, IL-8, MIG, EGFR, HLA-DR, IL1α, Keratin 8/18, MMP-1, MMP-9, PAI-1, SRB, tPA, uPA	The BE3C system models lung inflammation of the Th1 type, an environment that promotes monocyte and T cell adhesion and recruitment. This system is relevant for sarcoidosis and pulmonary responses to respiratory infections.
**CASM3C**		Coronary artery smooth muscle cells	Cardiovascular Inflammation, Restenosis	MCP-1, VCAM-1, TM, TF, uPAR, IL-8, MIG, HLA-DR, IL-6, LDLR, M-CSF, PAI-1, Proliferation, SAA, SRB	The CASM3C system models vascular inflammation of the Th1 type, an environment that promotes monocyte and T cell recruitment. This system is relevant for chronic inflammatory diseases, vascular inflammation and restenosis.
**HDF3CGF**		Dermal fibroblasts	Fibrosis, Chronic Inflammation	MCP-1, VCAM-1, ICAM-1, Collagen I, Collagen III, IP-10, I-TAC, IL-8, MIG, EGFR, M-CSF, MMP-1, PAI-1, Proliferation_72hr, SRB, TIMP-1, TIMP-2	The HDF3CGF system models wound healing and matrix/tissue remodeling in the context of Th1-type inflammation This system is relevant for various diseases including Fibrosis, Rheumatoid Arthritis, Psoriasis and stromal biology in tumors.
**KF3CT**		Keratinocytes + Dermal fibroblasts	Psoriasis, Dermatitis, Skin Biology	MCP-1, ICAM-1, IP-10, IL-8, MIG, IL-1α, MMP-9, PAI-1, SRB, TIMP-2, uPA	The KF3CT system models model cutaneous inflammation of the Th1 type, an environment that promotes monocyte and T cell adhesion and recruitment. This system is relevant for cutaneous responses to tissue damage caused by mechanical, chemical, or infectious agents as well as certain states of psoriasis and dermatitis.
**MyoF**		Lung fibroblasts	Fibrosis, Chronic Inflammation, Wound Healing, Matrix Remodeling	a-SM Actin, bFGF, VCAM-1, Collagen-I, Collagen-III, Collagen-IV, IL-8, Decorin, MMP-1, PAI-1, TIMP-1, SRB	The MyoF system models the development of myofibroblasts relevant to fibrotic diseases as well as other chronic inflammatory settings where tissue remodeling and fibrosis is involved including scleroderma, SLE, psoriasis and arthritis.
**Mphg**		Venular endothelial cells + Macrophages	Cardiovascular Inflammation, Restenosis, Chronic Inflammation	MCP-1, MIP-1α, VCAM-1, CD40, E-selectin, CD69, IL-8, IL-1α, M-CSF, sIL-10, SRB, SRB-Mphg	The Mphg System models chronic inflammation of the Th1 type and macrophage activation responses. This system is relevant to inflammatory conditions where monocytes play a key role including atherosclerosis, restenosis, rheumatoid arthritis, and other chronic inflammatory conditions.

doi:10.14573/altex.2203041s2

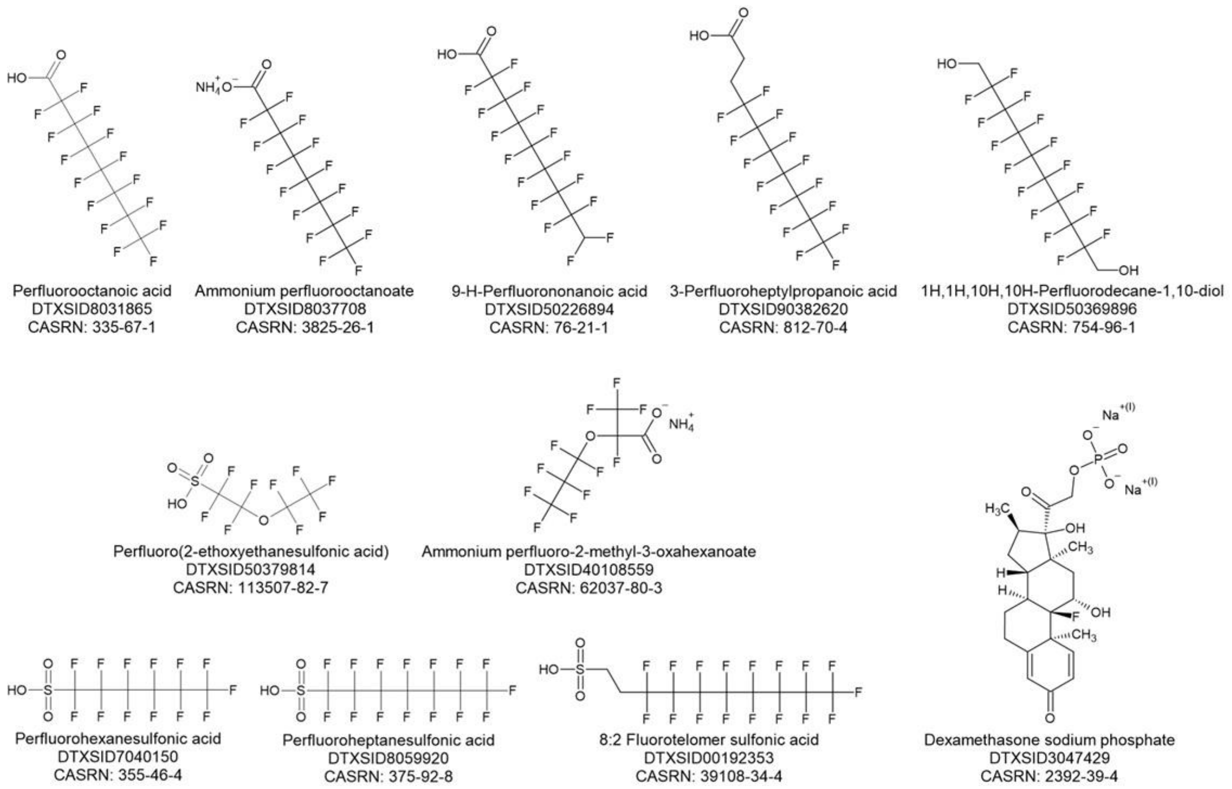

Fig. S1: Structures of chemicals in cluster 31

This is an Open Access article distributed under the terms of the Creative Commons Attribution 4.0 International license (http://creativecommons.org/licenses/by/4.0/), which permits unrestricted use, distribution and reproduction in any medium, provided the original work is appropriately cited.
